# An HSP90 cochaperone Ids2 maintains the stability of mitochondrial DNA and ATP synthase

**DOI:** 10.1186/s12915-021-01179-x

**Published:** 2021-11-11

**Authors:** Pei-Heng Jiang, Chen-Yan Hou, Shu-Chun Teng

**Affiliations:** 1 Department of Microbiology, College of Medicine, National Taiwan University, No. 1, Sec. 1, Jen-Ai Road, Taipei, 10051 Taiwan; 2 Center of Precision Medicine, National Taiwan University, Taipei, Taiwan

**Keywords:** Aging, Proteostasis, Mitochondria, Ids2, ATP synthase

## Abstract

**Background:**

Proteostasis unbalance and mitochondrial dysfunction are two hallmarks of aging. While the chaperone folds and activates its clients, it is the cochaperone that determines the specificity of the clients. Ids2 is an HSP90’s cochaperone controlling mitochondrial functions, but no in vivo clients of Ids2 have been reported yet.

**Results:**

We performed a screen of the databases of HSP90 physical interactors, mitochondrial components, and mutants with respiratory defect, and identified Atp3, a subunit of the complex V ATP synthase, as a client of Ids2. Deletion of *IDS2* destabilizes Atp3, and an α-helix at the middle region of Ids2 recruits Atp3 to the folding system. Shortage of Ids2 or Atp3 leads to the loss of mitochondrial DNA. The intermembrane space protease Yme1 is critical to maintaining the Atp3 protein level. Moreover, Ids2 is highly induced when cells carry out oxidative respiration.

**Conclusions:**

These findings discover a cochaperone essentially for maintaining the stability of mitochondrial DNA and the proteostasis of the electron transport chain—crosstalk between two hallmarks of aging.

**Supplementary Information:**

The online version contains supplementary material available at 10.1186/s12915-021-01179-x.

## Background

Aging is a process with a decline of organismal function and an increase in the risk of disease and death. Mitochondrial dysfunction is one of the nine hallmarks of aging [1] that can be produced by aging-associated mitochondrial DNA (mtDNA) mutations [2], reduced mitochondriogenesis [3], destabilization of the electron transport chain [4] complexes [4, 5], altered mitochondrial dynamics, and defective quality control by mitophagy [6]. In aged cells, the efficacy of the respiratory chain tends to diminish, thus increasing electron leakage and reducing ATP generation [7].

Animal decomposes food to obtain ATP through oxidative reactions mainly in mitochondria, where carbohydrates, proteins, and fats undergo a series of metabolic reactions collectively called cellular respiration. Cellular respiration oxidizes organic compounds to CO_2_ and H_2_O. The outer and the inner membranes (OM and IM) define two mitochondrial compartments: intermembrane space (IMS) and the central matrix. Oxidative phosphorylation (OXPHOS) is powered by the movement of electrons through the ETC (electron transport chain) complex I, II, III, and IV to generate a gradient of concentration of protons maintained in the IMS. The electrochemical proton gradient across the IM energizes ATP production by the complex V F_1_-F_0_ ATP synthase [8]. The F_0_ is a hydrophobic segment that spans the IM, which is the channel for the transport of protons from the IMS back into the matrix. The energy of this process also converts ADP and Pi into ATP in the F_1_ complex which resides in the matrix.

Mitochondria contain their genome. They divide by binary fission, similar to bacteria. Yeast mtDNA represents on average 15% of the total cellular DNA content [9] and consists mostly of linear molecules with varying lengths ranging from 75 to 150 kb [10]. In *Saccharomyces cerevisiae*, mtDNA encodes eight proteins, of which seven are subunits of the ETC and OXPHOS, and one is a ribosomal small subunit protein [11]. Interestingly, most mtDNA genes are conserved from yeast to humans [12]. mtDNA is considered a major target for aging-associated somatic mutations due to the oxidative microenvironment of mitochondria, lack of protective histones, and limited efficiency of the mtDNA repair mechanisms compared to those of nuclear DNA [13]. mtDNA integrity is maintained by its binding proteins [14, 15] and the excision repair system [16]. However, reactive oxygen species [17], drug, temperature, DNA polymerase γ mutations, disturbance of mitochondrial membrane potential (Δψ_m_), imbalance of proteases, and aging can change the integrity of mtDNA [18–22]. Under stress, mtDNA can also escape to intracellular or extracellular compartments, triggering intrinsic apoptosis and/or innate immune inflammatory response [23].

Chaperone systems play prominent roles in maintaining proteostasis. The heat shock protein (HSP) HSP70 and HSP90 systems are the main chaperone machinery for cellular protein folding [24] and misfolded proteins create pathological problems in different tissues. Protein aggregates are found in a variety of diseases, including type II diabetes, Parkinson’s, and Alzheimer’s diseases [25]. In addition to chaperones, the specificity of protein folding machines largely depends on cochaperones. They actively participate in various stages of the folding cycle [26]. Cochaperones bind to specific domains of HSPs to stabilize their conformation or modulate their functions. Besides, each cochaperone recruits its specific clients to the folding system [27]. Therefore, both chaperones and cochaperones control folding and proteostasis.

A previous study demonstrates crosstalk among three hallmarks of aging: deregulated nutrient sensing, loss of proteostasis, and mitochondrial dysfunction [28]. The HSP90 cochaperone Ids2 enhances HSP90’s chaperone activity during calorie restriction, enabling cells to maintain protein quality for sustained longevity. Ids2 can stimulate the ATPase activity of HSP90. Surprisingly, deletion of *IDS2* causes a severe growth defect in glycerol [28], but the detailed mechanisms are still unclear. Here, we developed a screening procedure from Hsc82 physical interactors to discover the first direct client of the HSP90-Ids2 chaperone complex and reveal the mechanism of Ids2 in maintaining mtDNA integrity and mitochondrial functions.

## Results

### Deletion of *HSC82* or *IDS2* causes a deficiency in mitochondrial functions

**Fig. 1:**
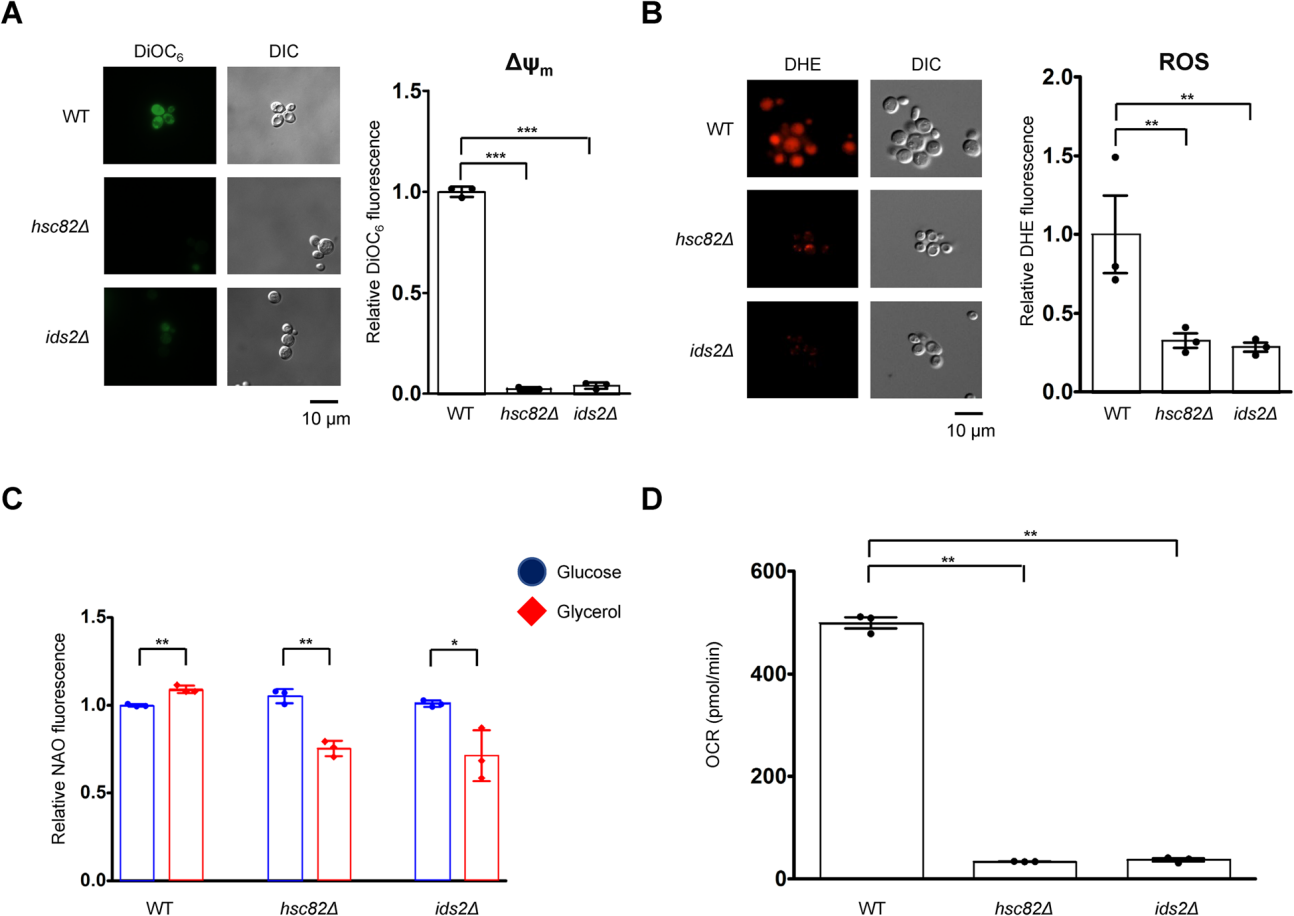
Mitochondrial function is reduced in *hsc82Δ* and *ids2Δ* cells. **A** Representative images and FACS analysis of the DiOC_6_-stained cells. Indicated strains were cultured in YEPG medium for 48 h. The Δψ_m_ was detected by FACS analysis after 30 min of DiOC_6_ staining. The DiOC_6_ fluorescence value was normalized to that of the wild-type strain. **B** Representative images and FACS analysis of the DHE-stained cells. Indicated strains were cultured in YEPD medium for 48 h. The intracellular levels of ROS generated in the indicated strains were examined by FACS analysis after 10 min of DHE staining. The DHE fluorescence value was normalized to that of the wild-type strain. **C** Mitochondrial mass of indicated strains cultured in YEPD or YEPG for 48 h were detected by FACS analysis after NAO staining. The NAO fluorescence value was normalized to that of the wild-type strain in the glucose medium. **D** The oxygen consumption rate of each strain was measured by a Seahorse XF Analyzers. The values are mean ± SD (*n* = 3) and compared by using Student’s *t* test. *, *p* value < 0.05; **, *p* value < 0.01; and ***, *p* value < 0.001. Points on bar graphs indicate individual data values from each biological replicate

We previously showed that Ids2 serves as a cochaperone of Hsc82, the major HSP90 in yeast, to maintain protein quality for cell longevity [28]. Interestingly, the growth defect of the *hsc82Δ* and *ids2Δ* cells in glycerol medium [28] was not observed in the *HSP82*, a paralog of *HSC82*, deleted cells (Additional File 1: Figures S1). We speculate that the growth defect of the *hsc82Δ* and *ids2Δ* cells may be caused by the loss of mitochondrial functions. To analyze the mitochondrial functions, we used fluorescent dyes to determine their Δψ_m_, production of ROS, and mitochondrial mass [29, 30]. Deletion of *HSC82* or *IDS2* decreased the Δψ_m_, as observed by microscopic imaging and flow cytometry analyses (Fig. 1A). The fluorescence of the ROS-sensitive probe DHE decreased significantly in stationary phase *hsc82Δ* and *ids2Δ* cells (Fig. 1B). To know whether the loss of Δψ_m_ and ROS production were due to the loss of mitochondrial function or the whole organelle, we used NAO staining to detect mitochondrial mass [31] in both fermentative (glucose) and respiratory (glycerol) growth. Under fermentative growth, the mitochondrial mass did not show a significant difference among wild-type, *hsc82Δ*, and *ids2Δ* stains. In respiratory growth, wild-type cells showed a slight increase in mitochondrial mass. However, the mitochondrial mass was decreased ~ 30% in *hsc82Δ* and *ids2Δ* cells (Fig. 1C), indicating that *hsc82Δ* and *ids2Δ* cells may have defective mitochondrial biogenesis [32]. The loss of Δψ_m_ implies that *hsc82Δ* and *ids2Δ* cells may have a defect in the respiratory chain [33]. To test this hypothesis, we tested oxygen consumption in wild-type, *hsc82Δ*, and *ids2Δ* cells. As expected, the oxygen consumption was decreased by 90% in *hsc82Δ* and *ids2Δ* cells, suggesting a severe defect in the respiratory chain (Fig. 1D). These results indicate that Hsc82 and Ids2 are essential for intact mitochondrial function.

### Ids2 maintains the stability of the assembly factor of cytochrome c oxidase and ATP synthase

**Fig. 2:**
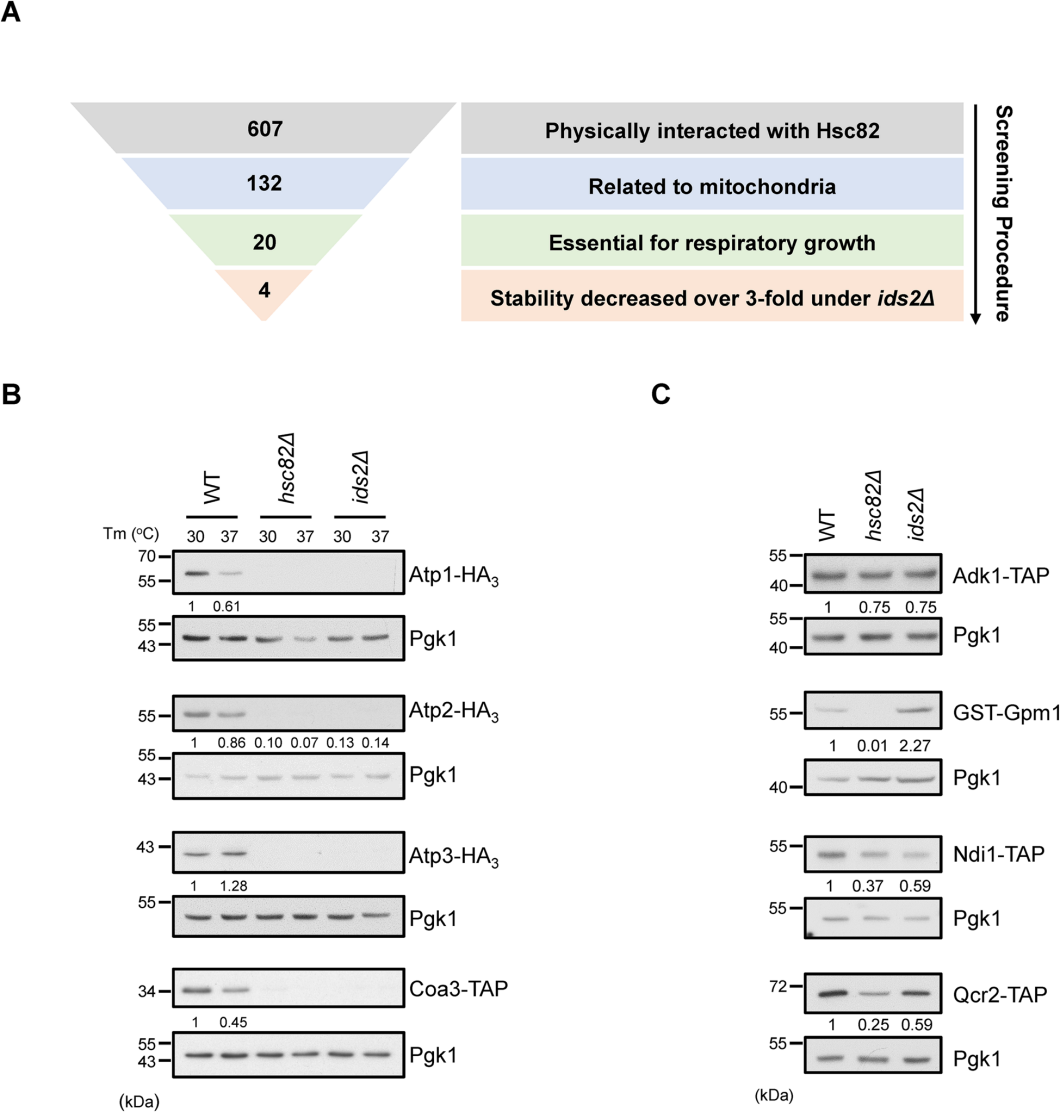
A screen for the clients regulated by the Hsc82-Ids2 chaperone complex. **A** A schematic diagram highlights the steps in the screening of the Hsc82-Ids2 clients. **B** Ids2 maintains the protein levels of four candidates in the ATP metabolic process. Overnight cells of the indicated strains were refreshed to OD = 0.5 and then transferred to 30 or 37 °C for 3 h. Total protein was extracted, and the western blot was hybridized with various antibodies. Pgk1 was served as a loading control. The numbers below are the means (*n* = 3) of the intensity ratios of HA/Pgk1 compared with that of the wild-type strain. **C** Ids2 does not control the stability of the other four candidates in the ATP metabolic process. Overnight cells of the indicated strains were refreshed to OD = 0.5 in 30 °C for 3 h. Western blotting was conducted as described above

To understand how the loss of Ids2 causes mitochondrial dysfunction and defective cellular respiration, we screened for the non-essential proteins that physically interact with Hsc82, proteins in mitochondria, and genes required for respiratory growth from SGD (Fig. 2A and Additional File 2: Table S1). According to these three criteria, 607 Hsc82 interacting proteins were narrowed down to 20 potential clients: Aco1, Acs1, Adk1, Atp1, Atp2, Atp3, Ccs1, Cor1, Coa3, Dcs1, Fum1, Gpm1, Mir1, Ndi1, Pet9, Por1, Qcr2, Sod1, Tuf1, and Vps1. According to Gene Ontology term analysis (Additional File 3: Table S2), most of the 20 potential clients were associated with ATP metabolic process (Adk1, Atp1, Atp2, Atp3, Gpm1, Ndi1, and Qcr2) and cellular respiration (Aco1, Cor1, Fum1, Ndi1, Pet9, Qcr2, and Sod1). The potential candidates were further tested for their protein stabilities in the *hsc82Δ* and *ids2Δ* background at 30 °C and 37 °C, except for three genes not available in both TAP- and GST-tagged libraries (Dsc1, Tub1 and Vps1). Only complex IV cytochrome c oxidase assembly factor Coa3 and complex V ATP synthase subunits Atp1, Atp2, and Atp3 were markedly downregulated (Fig. 2B), while others did not exhibit a substantial decrease in the *hsc82Δ* and *ids2Δ* cells (Fig. 2C and Additional File 1: Figures S2). The mRNA levels of these potential clients were not reduced in 30 °C (Additional File 1: Figures S3). These findings suggest that Ids2 maintains the stability of cytochrome c oxidase assembly factor and ATP synthase subunits, and the alteration of the protein amounts is not regulated at the transcriptional level.

### Suppression of Hsc82, Ids2, and Atp3 causes ETC damage, *petite* formation, and mtDNA loss

**Fig. 3:**
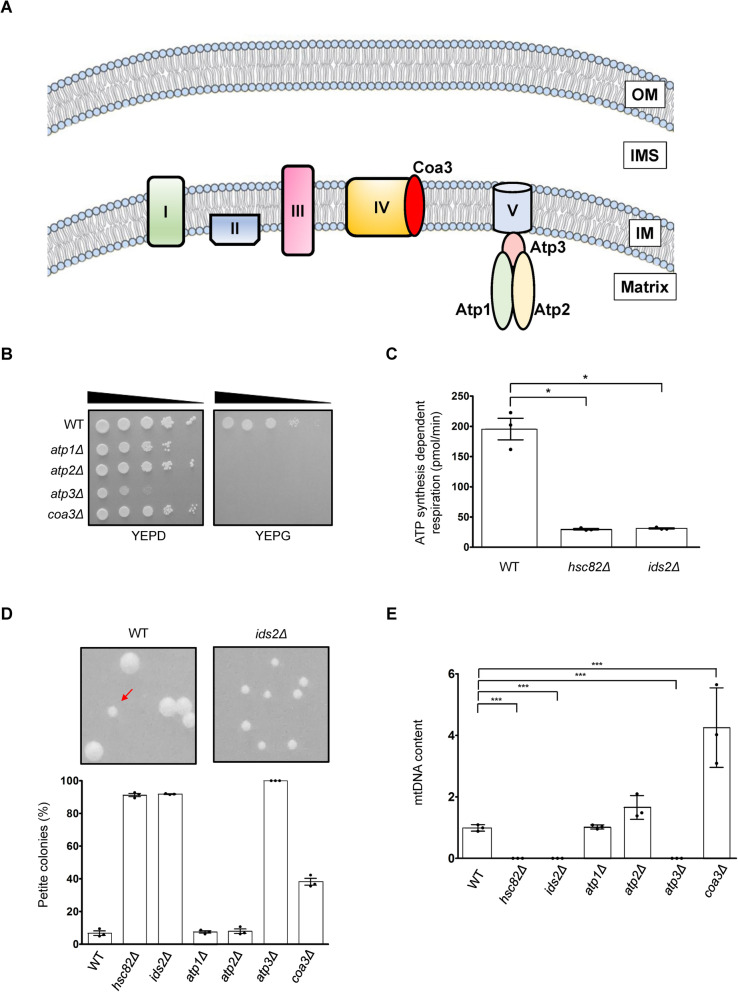
Inhibition of Hsc82, Ids2, and Atp3 triggers ETC damage, *petite* formation, and mtDNA loss. **A** A schematic diagram of potential clients’ locations in ETC. **B** Ten-fold serially diluted cells were grown under SC medium supplemented with 2% glucose or 3% glycerol. **C** ATP production of each yeast strain was measured by a Seahorse XF Analyzer. **D** Equal numbers of cells of the indicated strains were plated on glucose plates. A representative image of a petite colony in the wild-type strain (arrowhead) is shown. Almost all ids2Δ cells showed the petite phenotype. The percentage of petite colonies of the indicated strains is shown below. **E** The relative mtDNA content of the indicated strains was determined by quantitative PCR by measuring the amount of mitochondrial gene COX1 relative to the nuclear gene ACT1. The value was normalized to that of the wild-type strain. The values are mean ± SD (*n* = 3) and compared by using Student’s *t* test. *, *p* value **<** 0.05; **, *p* value **<** 0.01; and ***, *p* value **<** 0.001. OM, outer membrane; IMS, intermembrane space; IM, intermembrane. Points on bar graphs indicate individual data values from each biological replicate

ETC generates a proton gradient across the IM by pumping protons into the IMS, which drives the synthesis of ATP via coupling with OXPHOS with ATP synthase [34]. Dramatically, all of the four potential clients were located in the ETC (Fig. 3A), and deletion of each of them showed growth defect under glycerol condition (Fig. 3B). Besides, ATP production was significantly reduced in *hsc82Δ* and *ids2Δ* cells (Fig. 3C). To further prove the deficiency in ETC, we checked the respiration-deficient *petite* colonies and mtDNA in *atp1Δ*, *atp2Δ*, *atp3Δ*, and *coa3Δ* cells. Strikingly, only *atp3Δ*, but not *atp1Δ*, *atp2Δ,* and, *coa3Δ* showed severe *petite* phenotype (Fig. 3D) and complete loss of mtDNA (Fig. 3E), as observed in the *hsc82Δ* and *ids2Δ* cells. These results demonstrate that only *ATP3* deficiency causes similar mitochondrial phenotypes as the *hsc82Δ* and *ids2Δ* cells.

### Atp3 is a client of Ids2

**Fig. 4:**
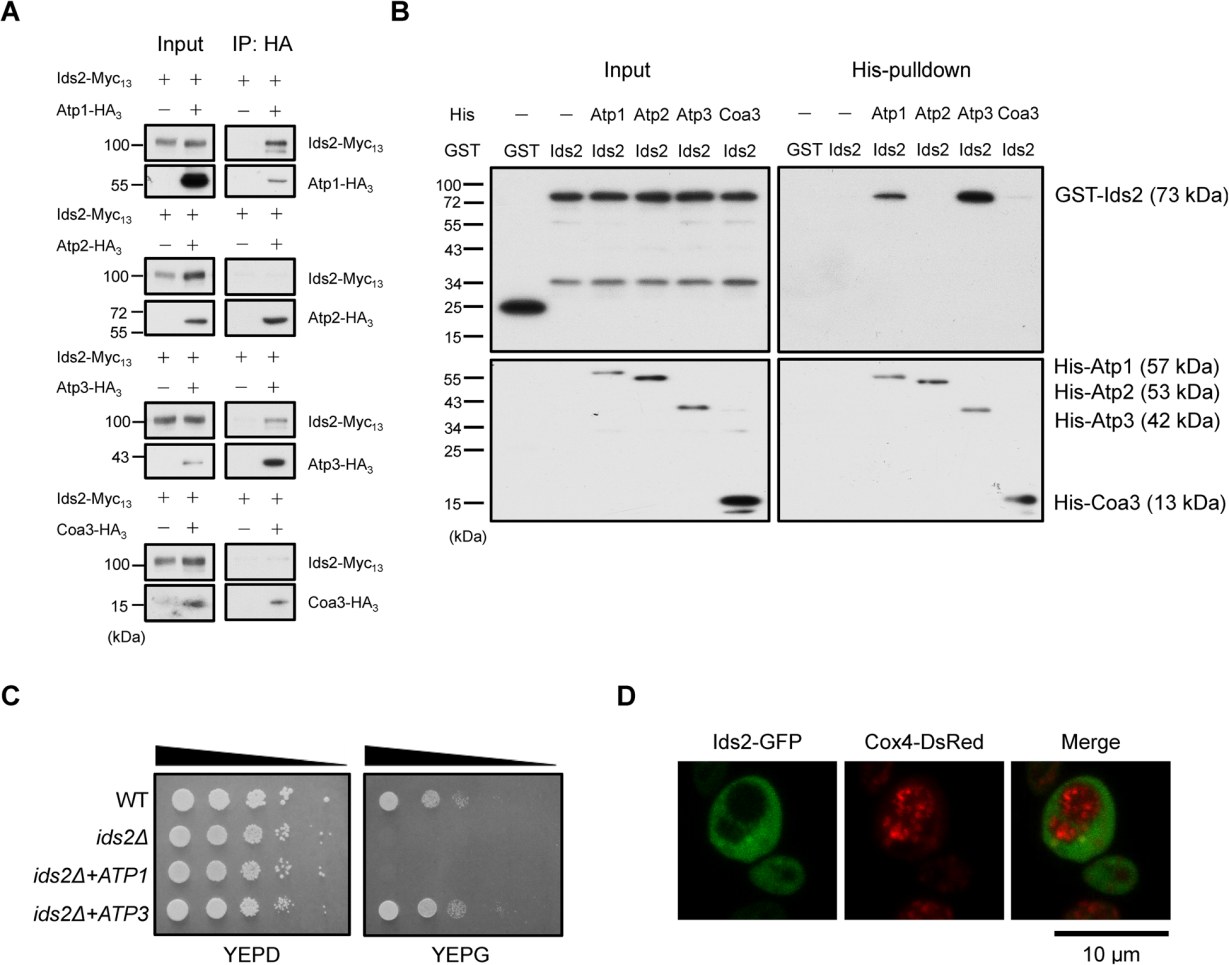
Ids2 interacts with Atp3 directly. **A** Co-immunoprecipitation assays were conducted using cells transformed with the pRS426-Ids2-Myc_13_ and pRS313-Atp1-HA_3_, pRS313-Atp2-HA_3_, pRS423-Atp3-HA_3_, or pRS426-Coa3-HA_3_ plasmids. Lysates were precipitated by an HA antibody and co-precipitated proteins were detected by a Myc antibody (*n* = 3). **B** Talon-bead-bound recombinant His6-Atp1, Atp2, Atp3, or Coa3 proteins were incubated with recombinant GST-tagged Ids2. The pulldown products were detected by the indicated antibodies (*n* = 3). **C** Ten-fold serially diluted cells were grown on SC medium plates without tryptophan supplemented with 2% glucose or 3% glycerol. **D** Confocal microscopic images show the localization of Ids2. W303 cells containing pRS426-*IDS2*-GFP and pHS12-*COX4*-DsRed (a mitochondria marker) were grown in SC medium supplemented with 3% glycerol for 48 h. Filters selective for the green fluorescence of GFP (left) or the red fluorescence of DsRed (middle) were used. Green and red fluorescence pictures merged are shown (right)

To confirm whether these four candidates are direct clients of Ids2, we analyzed their interactions with Ids2 both in vivo and in vitro. A co-immunoprecipitation assay showed that Atp1 and Atp3 interacted with Ids2 in vivo (Fig. 4A). Similar results were observed in in vitro pulldown assay where Atp1 and Atp3 displayed a mild and strong association with Ids2, respectively (Fig. 4B). To further define the major mitochondria-related client, we transformed the pRS414-*ATP1* or pRS414-*ATP3* plasmid into the *ids2Δ* cells to complement the glycerol growth defect. Only the Atp3, but not Atp1, could complement the deficiency of Ids2 (Fig. 4C). These data suggest that Atp3 may be a key client of Ids2 and the stability of other complex V candidates in *ids2Δ* cells might be reduced by the loss of Atp3. To test this possibility, we examined the protein stability of these candidates under the elimination of one of the candidates (Additional File 1: Figures S4A-C). Interestingly, deletion of *ATP3* reduced the protein level of Atp1 and Atp2, and deletion of *ATP1* or *ATP2* also decreased the level of Atp3. And a co-immunoprecipitation assay observed that Atp3 co-precipitated with Ids2 and HSP90 (Additional File 1: Figures S4D), indicating that Atp3-HSP90-Ids2 may form a ternary complex. All these results suggest that Atp3 may be a major client of Ids2. To understand where Ids2 interacts with Atp3 in cells, confocal microscopic images of Ids2 were captured. Ids2-GFP distributed in cytosol, which was separated from the mitochondrial IM protein Cox4-DsRed (Fig. 4D), implying that Ids2 may interact with Atp3 in the cytosol or near the mitochondrial OM.

### A middle motif in Ids2 recruits the N-terminal Atp3 to the folding system

**Fig. 5:**
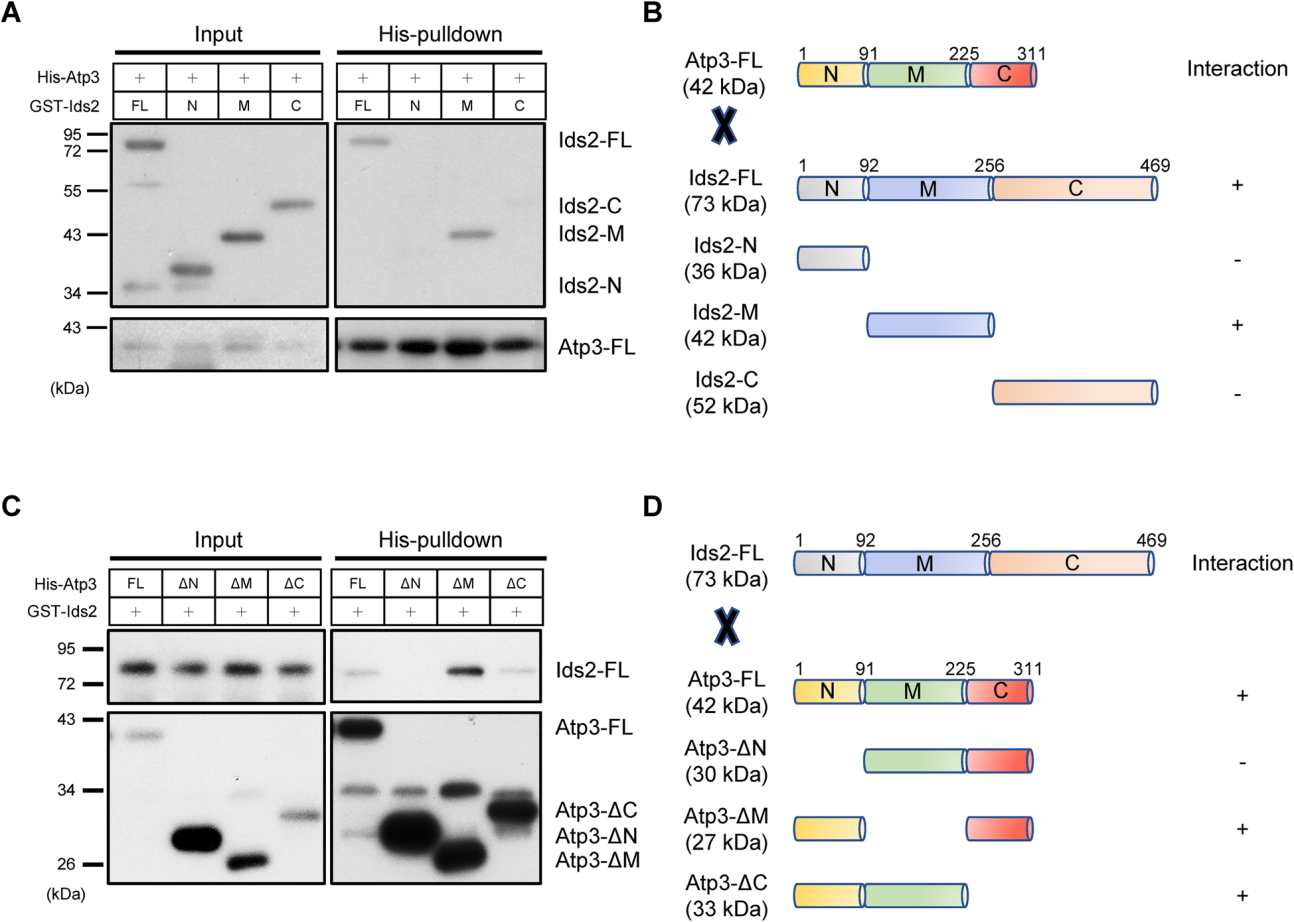
Domain requirement for the Ids2-Atp3 interaction. **A** Talon-bead-bound His_6_-Atp3 proteins were incubated with full-length or truncated Ids2 recombinant proteins (*n* = 3). The pulldown assay was conducted as described in Fig. 4. **B** A schematic diagram of Atp3 and various Ids2 truncations. **C** Talon-bead-bound full-length or truncated His_6_-Atp3 were incubated with full-length Ids2 recombinant proteins (*n* = 3). The pulldown assay was conducted as described above. **D** A schematic diagram of Ids2 and various Atp3 truncations

To understand how Ids2 recruits its client Atp3, multiple truncated proteins were tested for the Ids2-Atp3 interaction. Ids2 was truncated to N-terminal (amino acid 1-92), middle (aa 92-256) and C-terminal (aa 256-469) regions, and Atp3 was truncated to ΔN (1-91 deletion), ΔM (91-225 deletion), and ΔC (225-311 deletion) forms (Additional File 1: Figures S5). To test the direct interaction, we purified the recombinant proteins of each fragment from *E. coli*. The pulldown results indicated that the middle domain of the Ids2 interacts with the N-terminus of Atp3 (Fig. 5A–D). According to the previous study [28], Hsc82 also interacts with the middle region of Ids2, and the Hsc82-Ids2 interaction is regulated by the phosphorylation of Ids2 S148. However, a co-immunoprecipitation assay demonstrated that the interaction between Atp3 and Ids2 S148 mutants displayed no significant difference comparing with that of the Ids2 wild-type strain (Additional File 1: Figures S6A), implying that the motif interacting with Atp3 on Ids2 is distinct from that with Hsc82. These results identify the domain requirement for Ids2 to recruit Atp3 to the folding system.

**Fig. 6:**
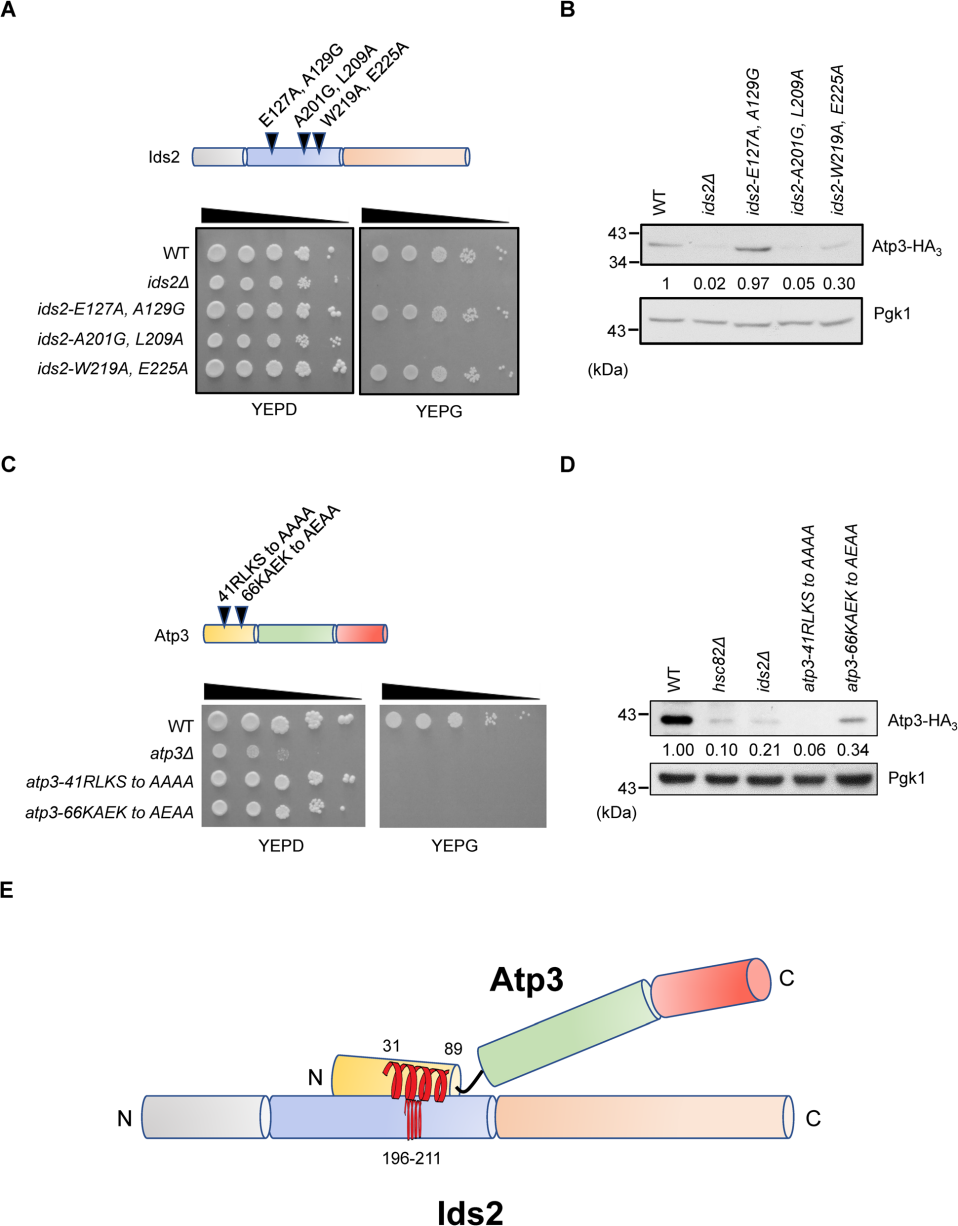
The Ids2 (196~211) and Atp3 (31~89) motifs are critical for the Ids2-Atp3 association. **A** Upper panel shows a schematic diagram of the Ids2 mutations. The ten-fold serially diluted *ids2* mutants were grown on SC plates supplemented with 2% glucose or 3% glycerol. **B** Indicated strains containing the pRS313-Atp3-HA_3_ plasmid were cultured overnight and then refreshed to OD = 0.5. Total proteins were extracted, and the Atp3-HA_3_ protein levels in these *ids2* mutants were detected by western blotting. **C** A schematic diagram of the Atp3 mutations and the ten-fold serial dilution of *atp3* mutants growing on SC plates supplemented with 2% glucose or 3% glycerol. **D** The mutated Atp3-HA_3_ p plasmids were transformed into wild-type cells. The Atp3 protein levels were detected by western blotting. The numbers below are the means (*n* = 3) of the intensity ratios of HA/Pgk1 compared with that of the wild-type strain. Tagged wild-type Atp3 in *hsc82Δ* or *ids2Δ* cells were used as negative controls. **E** A cartoon indicates that an Atp3 (31~89) motif interacts with an Ids2 (196~211) motif

To further define the motif of Ids2 that recruits its client to the folding system, the homologs of Ids2 were aligned and three conserved motifs in the middle region of Ids2 were subjected to mutagenesis and analysis (*ids2-E127A, A129G*, *ids2-A201G, L209A*, and *ids2-W219A, E225A*, Additional File 1: Figures S5A). Interestingly, the *ids2-A201G, L209A* cells exhibited growth defects under glycerol condition (Fig. 6A) and Atp1 and Atp3 were also markedly lost in the *ids2-A201G, L209A* cells (Fig. 6B and Additional File 1: Figures S6B). Analysis of the secondary structure by the CFSSP program [35] identified an α-helix spanning aa 196~211 of Ids2 which covers the mutated A201 and L209 residues (Additional File 1: Figures S5A), implying that this helix may be crucial for the Ids2 cochaperone to recruit its client.

To understand how an Ids2 client is attracted to the folding system, we next examined the Atp3 sequence. Alignment and secondary structure analyses of the Atp3 homologs identified an α-helix at the N-terminal region of Atp3 spanning from aa 31 to 89 (Additional File 1: Figures S5B). We generated two mutational strains to destroy the front (*atp3-41RLKS* to *AAAA*) and the rear (*atp3-66KAEK* to *AEAA*) motifs of the α-helix, respectively Additional File 1: Figures S5B). Both *atp3-41RLKS* to *AAAA* and *atp3-66KAEK* to *AEAA* cells exhibited growth defects under glycerol condition (Fig. 6C), but Atp3-41RLKS to AAAA protein was more unstable than Atp3-66KAEK to AEAA protein (Fig. 6D). In vitro pulldown assay of Ids2 and Atp3 mutants also showed a reduction of the Ids2-Atp3 interaction (Additional File 1: Figures S6C). These results imply that the front region of the N-terminal α-helix of Atp3 may be critical in Ids2-mediated Atp3 recruitment (Fig. 6E).

### Mitochondrial Yme1 and Pim1 proteases are essential for Atp3 quality control

**Fig. 7:**
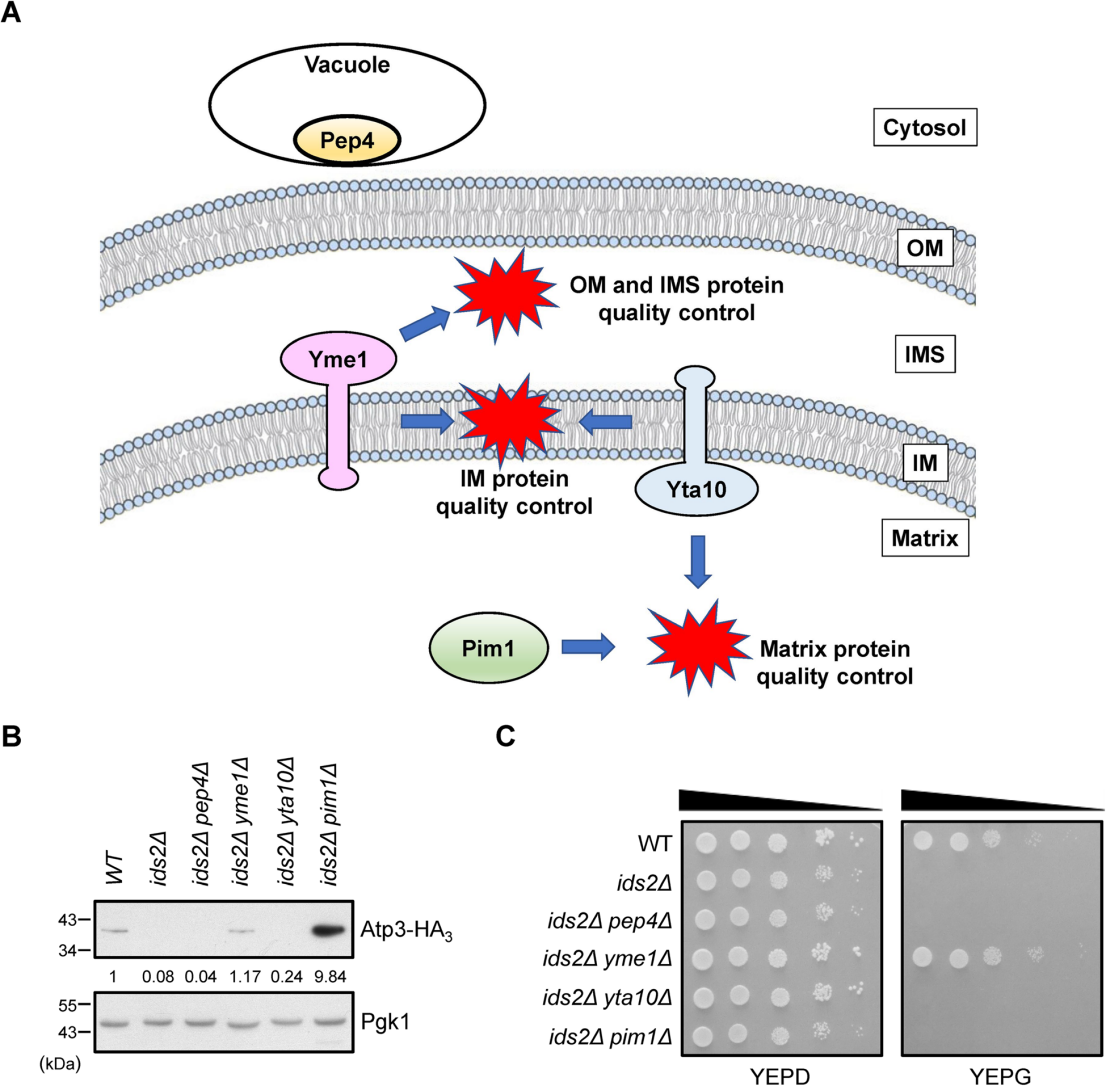
Protein quality control of Atp3. **A** A schematic diagram of various proteases in the cytosol and different compartments of mitochondria. **B** The protein levels of Atp3-HA_3_ in indicate strains were detected by western blotting. The numbers below are the means (*n* = 3) of the intensity ratios of HA/Pgk1 compared with that of the wild-type strain. **C** Ten-fold serially diluted cells were grown on YEP plates supplemented with 2% glucose or 3% glycerol

Proteostasis highly relies on chaperones and proteases to maintain proper folding and remove unfolded proteins. Cytoplasmic proteins can be degraded by the proteasome and the vacuolar proteolysis degradation pathways [36]. On the other hand, mitochondrial sub-compartments are under surveillance of ATP-dependent proteases for unfolded and unassembled proteins [17]. To understand the protease pathways controlling the quality control of Atp3 when the HSP90/Ids2 system fails to execute its folding function, we checked whether the vacuolar protease Pep4 [37], IMS/IM protease Yme1, IM/matrix protease Yta10 [38], and matrix protease Pim1 [39] modulate the Atp3 protein level in the absence of Ids2 (Fig. 7A). Interestingly, *YME1* or *PIM1* deletion could rescue the Atp3 level in the *ids2Δ* cells (Fig. 7B and Additional File 1: Figures S7B), suggesting that Yme1 and Pim1 control the amount of Atp3. And deletion of *IDS2* did not change the ratio of Atp3 inside mitochondria (Additional File 1: Figures S7C). However, only the *YME1* deletion could rescue the growth defect of *ids2Δ* cells in the glycerol medium (Fig. 7C). These results imply that undegraded Atp3 might not be able to recover the unbalanced mitochondrial function under the loss of protease Pim1. Because Atp3 is completely undetectable in the *ids2Δ* cells, to study the conformational difference of Atp3 in wild-type and *ids2Δ* cells, we collected undegraded Atp3 from *pim1Δ* and *ids2Δ pim1Δ* strains by immunoprecipitation followed with limited Proteinase K-mediated proteolysis. Interestingly, Atp3 in the *ids2Δ pim1Δ* strain was more sensitive to proteolytic digestion compared with that in the *pim1Δ* strain (Additional File 1: Figures S7D). These results suggest that absence of Ids2 may alter the conformation of Atp3, thereby rendering it more susceptible to proteolytic digestion.

### Ids2 is a mitochondria-dominant HSP90 cochaperone induced in the glycerol medium

Given that Ids2 is essential for mitochondria function, we asked whether Ids2 is a mitochondria-dominant HSP90 cochaperone induced under the requirement of oxidative respiration. A major cytoplasmic HSP90 cochaperone is Aha1, which also binds to the middle domain of HSP90 [40] and promotes Hsc82 ATPase activity as Ids2 [28]. We, therefore, compared the growth of wild-type, *hsc82Δ, ids2Δ,* and *aha1Δ* cells in the glycerol medium. Interestingly, *aha1Δ* cells did not exhibit a growth defect in glycerol (Additional File 1: Figures S8A). Atp3 was also stably maintained in the *aha1Δ* cells (Additional File 1: Figures S8B). In contrast, Ids2 was highly expressed in the glycerol medium (Additional File 1: Figures S8C). These results suggest that Ids2 is more important for mitochondrial function than cytoplasmic HSP90 cochaperone Aha1.

## Discussion

**Fig. 8:**
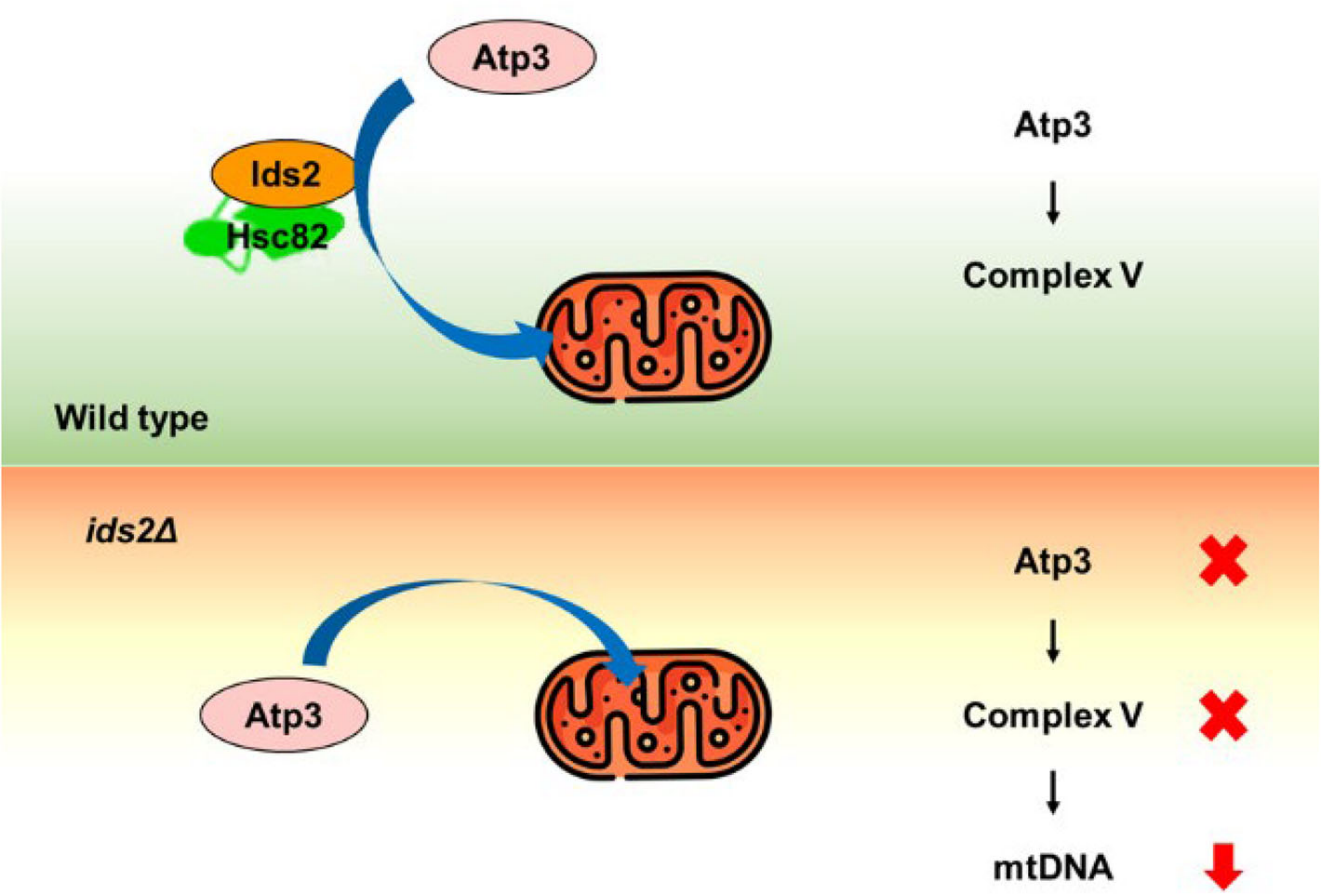
A proposed model of how the Hsc82-Ids2 chaperone complex regulates mtDNA and mitochondrial functions. Atp3 may be folded by the Hsc82-Ids2 chaperone complex before being imported into mitochondria. However, in *ids2Δ* cells, Atp3 may be degraded and incompletely assembled complex V may prevent protons from flowing across the IM. To overcome the stress of continuously increasing proton concentration in IMS, mitochondria may eliminate mtDNA to disrupt ETC and stop proton accumulation

The proton-pumping complexes of the ETC produce and maintain an electrochemical proton gradient across the membrane that energizes ATP production by ATP synthase. ATP synthase consists of the IM-bound F_0_ region and the matrix-exposed F_1_ region. Atp3, the γ subunit of ATP synthase, is the central subunit connecting F_1_ and F_0_ for the integrity of the ATP synthase [41]. Here we define a proteostasis system of the ATP synthase. The absence of the Hsc82-Ids2 complex may lead to Atp3 misfolding (Fig. 8). Without Atp3, ATP synthase assembly is disrupted, which leads to proton accumulation in the IMS. A continuously increasing proton concentration in the IMS may disturb the IM lipid bilayer, rise mitochondrial membrane permeability, and eventually induce a non-specific pore across the IM which could permit free distribution of mtDNA [42, 43].

Most yeast mitochondrial proteins are encoded by nuclear genes, synthesized in cytoplasmic ribosomes, and then imported into the organelle [44]. The newly synthesized polypeptides first contact the mitochondrial OM via Hsp70/Hsp90 chaperones and the Tom70 receptor to deliver preproteins into mitochondria [45]. Given that Ids2 is a cytoplasmic cochaperone and deletion of *IDS2* did not change the mitochondrial ratio of Atp3, we speculate that Ids2 may participate in Atp3’s folding before mitochondrial import. Indeed, clients need to be unfolded before mitochondrial import and we cannot exclude the possibility that Ids2 may participate in the unfolding step of Atp3 right before its mitochondrial import. Without the assistance of the Hsc82/Ids2 complex, Atp3 may be subjected to protein degradation.

Atp1 and Atp2 are components of the F_1_ catalytic head, and Atp3 is the F_1_ central stalk [46]. Loss of any subunits may block the assembly of ATP synthase and lead to aggregation of misassembled subunits [47]. Interestingly, interference of Atp3 destabilizes Atp1 and Atp2, and depletion of Atp1 or Atp2 also decreases the protein level of Atp3. Although the complement of Atp3 in *ids2Δ* cells restoring the respiration defect suggests that Atp3 may be a key protein of the complex V, these subunits may affect each other in assembly and stability.

Many evidence showed that the human HSP90 family is directly involved in mitochondria protein quality control and critical in cellular homeostatic [48]. Several studies demonstrated that the chaperone activity of the HSP90 family is correlated with OXPHOS and OXPHOS-coupled ATP synthesis [49, 50]. Recent researches in TRAP1, a mitochondria-specific HSP90 paralog, using IP-MS analysis, discovered how TRAP1 contributes to regulating OXPHOS and mitochondrial homeostasis [51]. Conversely, there is no mitochondria-specific HSP90 in yeast and Hsc82 exists in both cytoplasm and mitochondria [52]. By screening yeast Hsc82 interactors, we found that Hsc82-Ids2 is specifically required for F_1_-ATPase synthase assembly. Since Ids2 only exists in the cytoplasm, we speculate that Hsc82-Ids2 modulates ATP synthase assembly [53] in the cytoplasm through Atp3.

Ids2 binds Hsc82 and enhances Hsc82’s ATPase activity [28]. This scenario is very similar to another cytoplasmic HSP90 cochaperone Aha1 [40]. Under the heat shock condition, Aha1 is highly expressed to help HSP90 to protect their cytoplasmic clients [54]. Here we uncover that Ids2 is upregulated in the glycerol condition. Therefore, we propose that different environmental stresses may stimulate distinct cochaperones to execute their related regulation (Figure S9), and Ids2 cochaperone is specifically activated for protein quality control and mtDNA maintenance during OXPHOS.

## Conclusion

The proton-pumping complexes at the mitochondrial IM produce an electrochemical proton gradient across the membrane that energizes ATP synthase-mediated ATP production. But how cells maintain ATP synthase is still elusive. Here we define a proteostasis system of ATP synthase. The Hsc82-Ids2 complex is critical for Atp3’s stability. The absence of Hsc82-Ids2 leads to misfolding and degradation of Atp3. And Atp3 or ATP synthase dysfunction can cause proton accumulation in the IMS, which ultimately induces leakage of mtDNA [42, 43]. These findings reveal a mechanism of how a cytoplasmic cochaperone protects a unique client and further safeguards mitochondrial genome integrity.

## Methods

### Plasmids and yeast strains

*S. cerevisiae* W303 was used as the parental and wild-type strain. Standard genetic and cloning methods were used for all constructions [55]. Deletion mutants and TAP-tagged strains were generated by the double crossover of various selection marker fragments amplified from the Yeast Deletion or TAP-tagged Library (Horizon Discovery) to their wild-type locus. GST-tagged plasmids were obtained from the Yeast GST-tagged ORF Library (Horizon Discovery). The Myc- or HA-tagged strains were created by integrating the Myc_13_ or HA_3_ tag in-frame downstream of specific genes in the genome. The liquid yeast growth media were rich medium (YEP, 1% yeast extract, 2% peptone) or synthetic complete (SC) medium containing either 2% glucose or 1% glycerol with 1% raffinose. Cells were refreshed to the mid-log phase and harvested for the follow-up assays.

For tagged protein expression or phenotype complementation tests, genes were usually inserted into pRS313 or pRS314 [56]. However, genes with low expression levels were inserted into 2 μ plasmid pRS426 or pRS424. For chromosomal mutations, genes were introduced into pRS306. The pRS306 plasmids were linearized by the appropriate restriction enzymes and transformed into the wild-type strain. The pop-out mutants were selected by 0.01% 5-fluoroorotic acid. All yeast strains, primer sequences, and constructs used in this study are listed in Additional File 4: Table S3.

### Flow cytometry analysis of mitochondrial functions

To analyze the Δψ_m_, overnight cultured cells in YEP glucose (YEPD) medium were transferred to YEP glycerol (YEPG) for 48 h and washed with phosphate-buffered saline (PBS). Next, 1 × 10^7^ cells were resuspended in 1 ml YEPG with 175 nM DiOC_6_. The cell suspension was incubated for 30 min in the dark and washed with PBS. The stained cells were diluted to 1 × 10^6^ cells/ml and analyzed by FACSCalibur using the FL1 channel without compensation.

To analyze the ROS production, cells were cultured in YEPD for 48 h and washed with PBS. Next, 1 × 10^7^ cells were resuspended in 100 μl of PBS, and dihydroethidium (DHE) (Molecular Probes) was added to a final concentration of 50 μM. The cell suspension was incubated for 10 min in the dark and washed with PBS. The stained cells were diluted to 1 × 10^6^ cells/ml and analyzed by FACSCalibur using the FL3 channel without compensation.

To analyze the mitochondrial mass, cells were cultured in YEPD or YEPG for 48 h and stained for 10 min in 100 nM of nonyl acridine orange (NAO). Stained cells were detected by FACS analysis using the FL1 channel without compensation.

### Fluorescence microscopy

To analyze the Δψ_m_, cells of indicated strains were cultured in YEPG for 48 h and visualized directly or after 30 min staining with 175 nM DiOC_6_. To analyze the ROS production, cells of indicated strains were cultured in YEPD for 48 h and visualized directly or after 10 min staining with 50 μM DHE. All fluorescence images were captured using an Upright Fluorescence microscope (Zeiss AxioImager. M1). To analyze Ids2 localization, W303 cells containing pRS426-*IDS2*-GFP and pHS12-*COX4*-DsRed (a mitochondria marker) were grown in SC medium supplemented with 3% glycerol for 48 h and visualized directly. Fluorescence images were captured using a confocal microscope (Zeiss LSM880).

### Measurement of cellular oxygen consumption and ATP production

Cellular oxygen consumption rates (OCR) were measured with an XFp Extracellular Flux Analyser and the corresponding Seahorse Wave Desktop Software (Seahorse Bioscience). Yeast cells were refreshed in SC medium supplemented with 2% glucose and seeded in Seahorse XFp plates coated with Cell-tak (Invitrogen) at 1 × 10^6^ cells per well. Basal OCR was measured for 15 min at 30 °C. ATP production was calculated by the difference of OCR after 90 min treatment of the ATP synthase inhibitor triethyltin.

### *Saccharomyces* Genome Database and Gene Ontology term analyses

The *Saccharomyces* Genome Database (SGD; http://www.yeastgenome.org/) integrates functional information of budding yeast genes. Potential clients were chosen by the intersection of three pieces of information in SGD: physical interaction proteins of Hsc82, Gene Ontology term mitochondrion, and phenotype of respiratory growth. Essential genes were ruled out in this study because *IDS2* deletion is not lethal. Gene Ontology term analyses were conducted with the online resource GO Term Finder supplied in SGD [57, 58].

### Quantitative PCR (qPCR)

To identify mRNA expression level, indicated yeast cells were refreshed in YEPD at 30 or 37 °C for 3 h and collected for RNA purification. RNA was extracted using TRIzol reagent (Invitrogen). Complementary DNA (cDNA) was synthesized using SuperScript III Cells Direct cDNA Synthesis Kit (Invitrogen). Quantitative reverse transcription PCR (qRT-PCR) was performed on a BioRad CFX Connect Real-Time PCR Detection System.

Cellular DNA was extracted from the indicated strains to detect mtDNA copy numbers using qPCR. The relative mtDNA copy number was calculated using qPCR signals from the mtDNA *COX1* relative to those from the nuclear DNA *ACT1*.

### Glycerol viability test

Yeast cells were grown overnight at 30 °C. Overnight cultures were inoculated into fresh YEP or SC glucose medium and grown to exponential phase (OD_600_ = 0.5). Ten-fold serial dilutions of indicated strains were spotted onto SC plates with 2% glucose or 3% glycerol and incubated at 30 °C for 2 to 3 days.

### Co-immunoprecipitation assay

W303 strains containing C-terminal tagged plasmids (pRS313-*ATP1*-*HA*_*3*_, pRS313-*ATP2*-*HA*_*3*_, pRS423-*ATP3*-*HA*_*3*_, or pRS426-*COA3*-*HA*_*3*_) and pRS423-*IDS2*-*Myc*_*13*_ or pRS426-*IDS2*-*Myc*_*13*_ were grown to OD = 1 in SC medium supplemented with 3% glycerol. Pellets were resuspended in the lysis buffer (50 mM NaCl, 0.1% NP-40, 150 mM Tris-HCl, pH 8.0) supplemented with protease inhibitors (Roche). Cells were broken by a *FastPrep*-24 5G Homogenizer (MP biomedical) and supernatants were collected after centrifugation. The supernatants were mixed with either anti-HA (Roche) or anti-Myc (Roche) antibodies, and followed by incubation with protein G Sepharose beads. Immunoprecipitates were washed four times with the lysis buffer and then eluted by boiling in sample buffer. Samples were resolved by 10% SDS-PAGE and analyzed by western blotting using the appropriate antibodies.

### His-tagged metal affinity purification assay

pET28a or pGEX4T-1 plasmid was inserted with desired genes and transformed into the BL21 (DE3) tRNA strain. Cells were grown in LB media at 37 °C overnight, refreshed to OD = 0.6–0.8, and induced with 0.1 mM Isopropyl-β-D-thiogalactoside (IPTG, Sigma-Aldrich) at 16 °C for 6 h. Cells were collected by centrifugation and lysed with the His-lysis buffer (300 mM NaCl, 50 mM NaH_2_PO_4_, 10 mM imidazole, 3 mM β-mercaptoethanol, 0.5% NP-40, 10% glycerol, pH 8.0) or the GST-lysis buffer (300 mM NaCl, 2.7 mM KCl, 10 mM NaH_2_PO_4_, 1.8 mM KH_2_PO_4_, 3 mM β-mercaptoethanol) supplemented with 1× Protease Inhibitor Cocktails (Roche), 1 mM PMSF, and 1 mg/ml lysozyme (Sigma-Aldrich). After sonication, lysates were centrifuged, and supernatants were incubated with Talon Superflow or Glutathione Sepharose 4B resin (GE HealthCare) for 2 h at 4 °C. Beads were washed with the His-washing buffer (300 mM NaCl, 50 mM NaH_2_PO_4_, 20 mM imidazole, 10% glycerol, pH 8.0) or the GST-washing buffer (300 mM NaCl, 2.7 mM KCl, 10 mM NaH_2_PO_4_, 1.8 mM KH_2_PO_4_). For GST-tagged proteins, the beads were eluted by the elution buffer (50 mM Tris-HCl, pH 8.0, 10 mM reduced glutathione) and concentrated by Amicon Ultra-0.5 centricon (Millipore).

Talon bead-attached His-tagged proteins were incubated with 5 μg GST proteins in the incubation buffer (20 mM Tris-HCl, pH 7.4, 100 mM NaCl, 0.1% NP-40) supplemented with 1× Protease Inhibitor at 4 °C for 1 h. After washing with the washing buffer (20 mM Tris-HCl, pH 7.4, 100 mM NaCl, 50 mM imidazole, 0.1% NP-40), sample buffer was added to the beads, and samples were incubated at 95 °C for 5 min for immunoblotting.

### Limited proteolysis

W303 *pim1Δ* and *ids2Δ pim1Δ* strains containing pRS314-*ATP3*-*HA*_*3*_ were grown to log phase. All subsequent manipulations were conducted at 4 °C. Cell lysis was performed using glass beads in a native lysis buffer (10 mM Tris-HCl, pH 8.5, 50 mM NaCl, 15 mM MgCl_2,_ and 5 mM DTT). Lysates were incubated with 1 ml pre-equilibrated anti-HA sepharose beads. After washing, the beads were resuspended in 20 μl lysis buffer with 400 μg/ml Proteinase K. Proteolytic digestion was conducted on ice for the indicated period (0~40 min). The reaction was stopped by adding 2 μl of PMSF (10 mM) and Atp3 fragments were detected by western blotting.

### Mitochondria isolation

W303 *pim1Δ* and *ids2Δ pim1Δ* strains containing pRS414-Atp3-HA_3_ were grown in SC medium supplemented with 1.5% glycerol and 1.5% raffinose. Cells were treated with zymolyase and lysed with a Dounce homogenizer, and cellular compartments were isolated by differential centrifugation as described in Yeast Protocol [59].

## Supplementary Information

**Additional file 1: Figures S1-S8.** **Figure S1.** *HSC82* deleted cells exhibit respiratory defects and comparable expression of Hsp82. **Figure S2**. Nine candidates in the cellular respiration process do not exhibit drastic alteration in protein stability in *hsc82Δ* and *ids2Δ* cells. **Figure S3.** mRNA expression levels of the potential clients regulated by the Hsc82-Ids2 chaperone complex. **Figure S4.** Deletion of a potential client can disturb the protein stability of other candidates and HSP90-Ids2-Atp3 forms a ternary complex. **Figure S5.** Sequence alignments and secondary structure analyses of the Ids2’s and Atp3’s homologs. **Figure S6.** The Ids2-Atp3 interaction and the Atp3 stability under various *ids2* mutant backgrounds. **Figure S7.** Respiratory growth, Atp3 levels, Atp3 import, and Atp3 folding in various protease or *IDS2* deletional strains. **Figure S8.** Ids2 plays a dominant role for mitochondria under respiratory growth. **Figure S9.** A proposed model describes that two Hsc82 cochaperones, Ids2 and Aha1, may split their works under different environmental conditions.

**Additional file 2: Table S1.** Screen results from SGD database.

**Additional file 3: Table S2.** GO terms analysis of 20 candidate genes.

**Additional file 4: Table S3.** Yeast strains, plasmids, and primer sets used in this study.

**Additional file 5.** Images of the full immunoblots.

## Data Availability

All supporting data in this study are provided in the main article or the associated additional files.

## Abbreviations

DHE: Dihydroethidium
DiOC_6_: 3,3′-Dihexyloxacarbocyanine iodide
ETC: Electron transport chain
HSP: Heat shock protein
IM: Inner membranes
IMS: Intermembrane space
NAO: Nonyl acridine orange
OM: Outer membranes
OXPHOS: Oxidative phosphorylation
ROS: Reactive oxygen species
