# Supplementary material for: An HSP90 cochaperone Ids2 maintains the stability of mitochondrial DNA and ATP synthase
Source: BMC Biol. 2021 Nov 11;19:242. doi: 10.1186/s12915-021-01179-x (PMC8582188; doi:10.1186/s12915-021-01179-x)
Supplement: Supplementary file 1 — Additional file 1: Figures S1-S8. Figure S1. HSC82 deleted cells exhibit respiratory defects and comparable expression of Hsp82. Figure S2. Nine candidates in the cellular respiration process do not exhibit drastic alteration in protein stability in hsc82Δ and ids2Δ cells. Figure S3. mRNA expression levels of the potential clients regulated by the Hsc82-Ids2 chaperone complex. Figure S4. Deletion of a potential client can disturb the protein stability of other candidates and HSP90-Ids2-Atp3 forms a ternary complex. Figure S5. Sequence alignments and secondary structure analyses of the Ids2’s and Atp3’s homologs. Figure S6. The Ids2-Atp3 interaction and the Atp3 stability under various ids2 mutant backgrounds. Figure S7. Respiratory growth, Atp3 levels, Atp3 import, and Atp3 folding in various protease or IDS2 deletional strains. Figure S8. Ids2 plays a dominant role for mitochondria under respiratory growth. Figure S9. A proposed model describes that two Hsc82 cochaperones, Ids2 and Aha1, may split their works under different environmental conditions. [file 12915_2021_1179_MOESM1_ESM.docx]

**ADDITIONAL FILES**


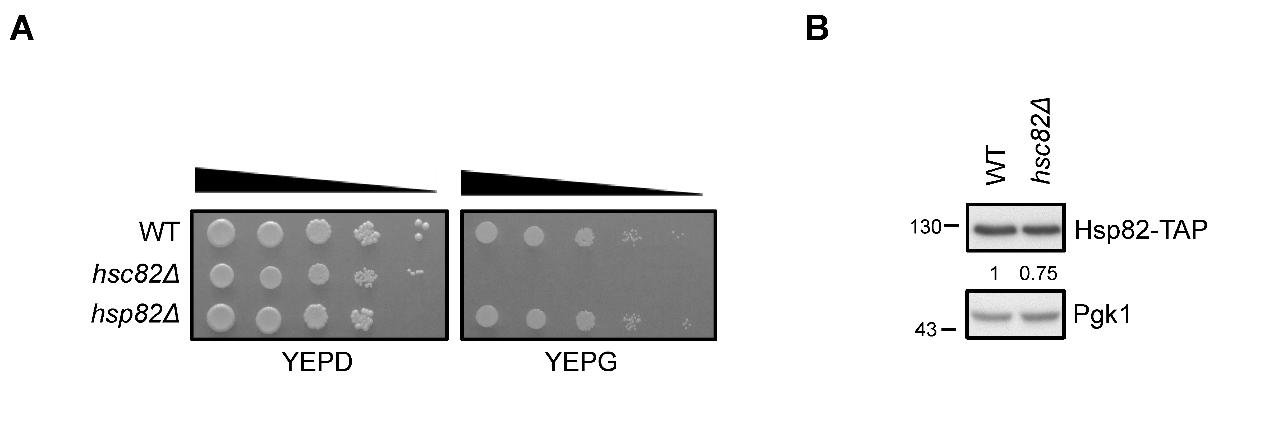


**Figure S1. *HSC82* deleted cells exhibit respiratory defects and comparable expression of Hsp82.**

**(A)** Ten-fold serially diluted cells were grown on YEP plates supplemented with 2 % glucose or 3 % glycerol. **(B)** Indicated strains with TAP-tagged Hsp82 were cultured overnight and then refreshed to OD = 0.5 in 30 ^o^C. Total protein was extracted, and the western blots were hybridized with TAP antibodies. Pgk1 was served as a loading control. The numbers below are the intensity ratios of protein level/Pgk1 compared with that of the wild-type strain.

**
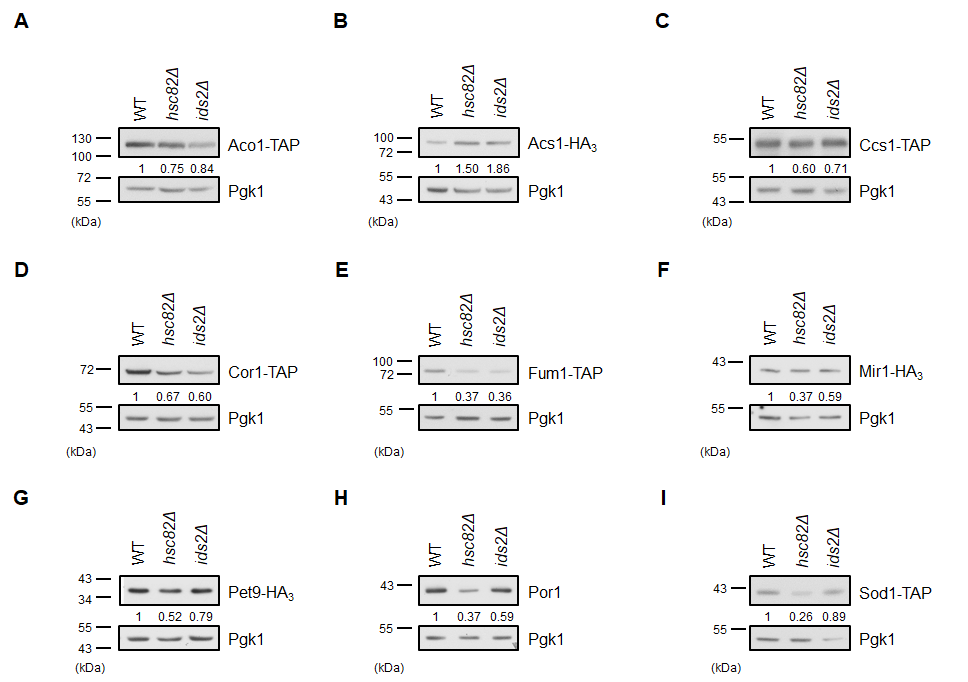
**

**Figure S2. Nine candidates in the cellular respiration process do not exhibit drastic alteration in protein stability in *hsc82Δ* and *ids2Δ* cells.**

**(A-I)** Indicated strains with various chromosomal tagging on nine candidates in the cellular respiration process were cultured overnight and then refreshed to OD = 0.5 in 30 ^o^C. Total protein was extracted, and the western blots were hybridized with appropriate antibodies. Pgk1 was served as a loading control. The numbers below are the intensity ratios of protein level/Pgk1 compared with that of the wild-type strain.


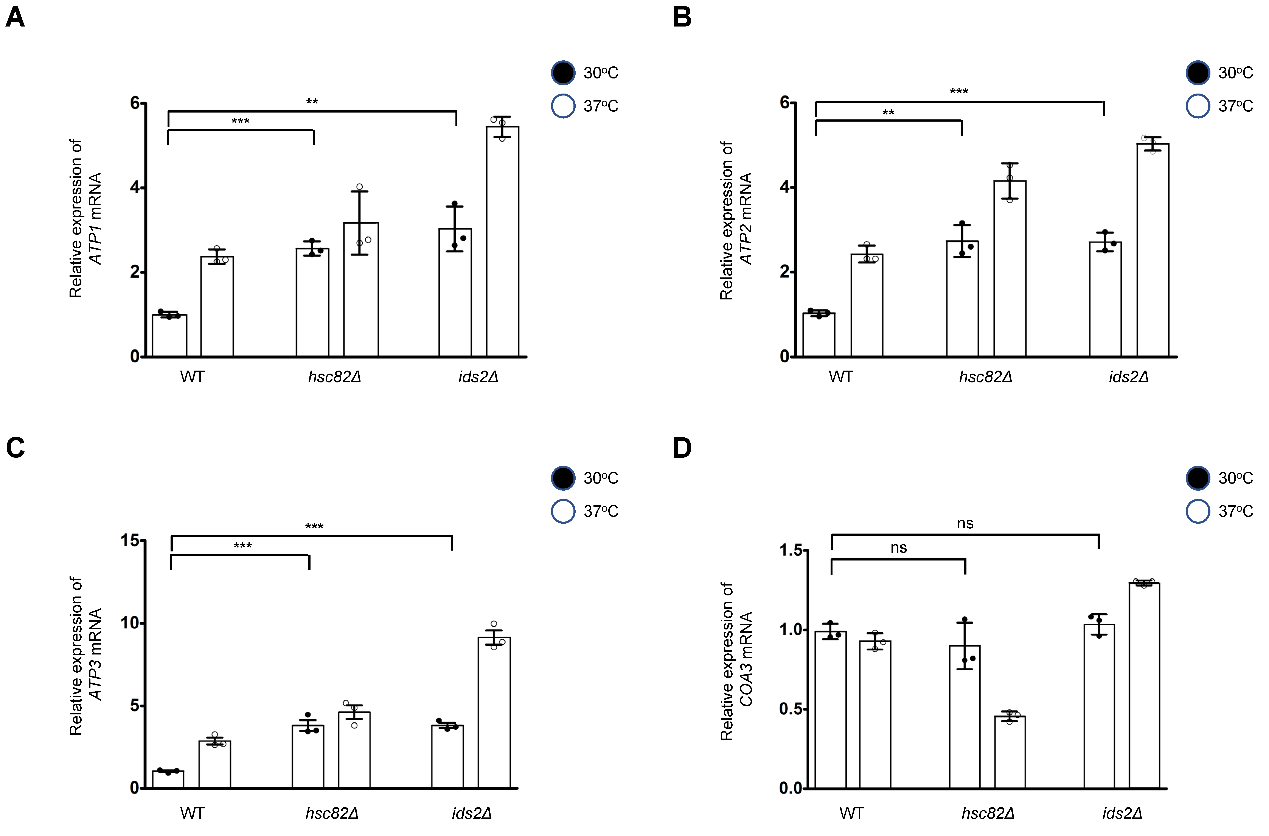


**Figure S3. mRNA expression levels of the potential clients regulated by the Hsc82-Ids2 chaperone complex.**

**(A-D)** Overnight cells of indicated strains were refreshed to OD = 0.5 and then transferred to 30 or 37 ^o^C for 3 hr. The mRNA levels of clients were determined by quantitative RT-PCR relative to a housekeeping gene, *ACT1*. The value was normalized to that of the wild-type strain treated at 30 °C. The values are mean ± SD (n = 3). The values of the indicated strains at 30 ^o^C were compared by using the Student’s t-test. *, *p*-value < 0.05; **, *p-*value < 0.01; and ***, *p*-value < 0.001. Points on bar graphs indicate individual data values from each biological replicate.


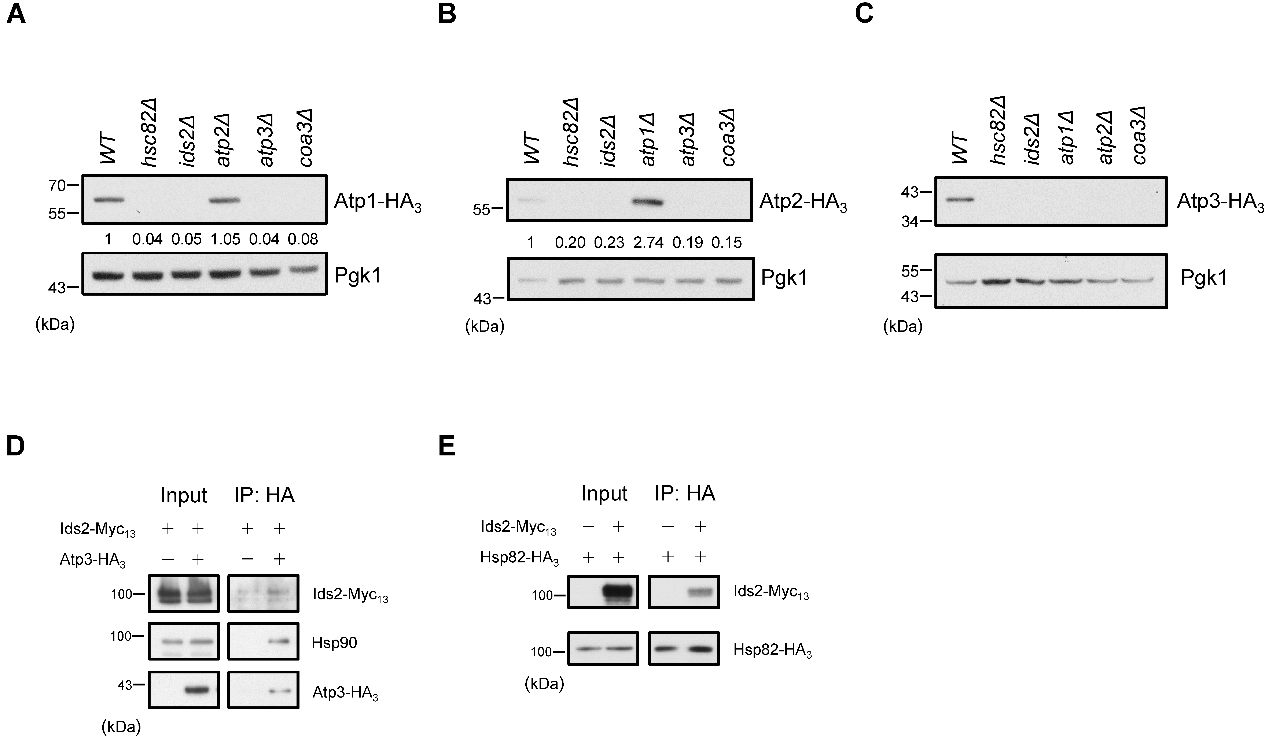


**Figure S4. Deletion of a potential client can disturb the protein stability of other candidates and HSP90-Ids2-Atp3 forms a ternary complex.**

**(A-C)** Indicated strains were cultured overnight and then refreshed to OD = 0.5. Total proteins were extracted, and the western blot was hybridized with an HA antibody. Pgk1 was served as a loading control. The numbers below are the intensity ratios of protein level/Pgk1 compared with that of the wild-type strain. **(D)** Co-immunoprecipitation assays were conducted using cells transformed with the pRS426-Ids2-Myc_13_ and pRS423-Atp3-HA_3_ plasmids. Lysates were precipitated by an HA antibody and co-precipitated proteins were detected by Myc and HSP90 antibodies. **(E)** Co-immunoprecipitation assays were conducted using cells with the chromosomally tagged Ids2-Myc_13_ and Hsp82-HA_3_. Lysates were precipitated by an HA antibody and co-precipitated proteins were detected by a Myc antibody.

**
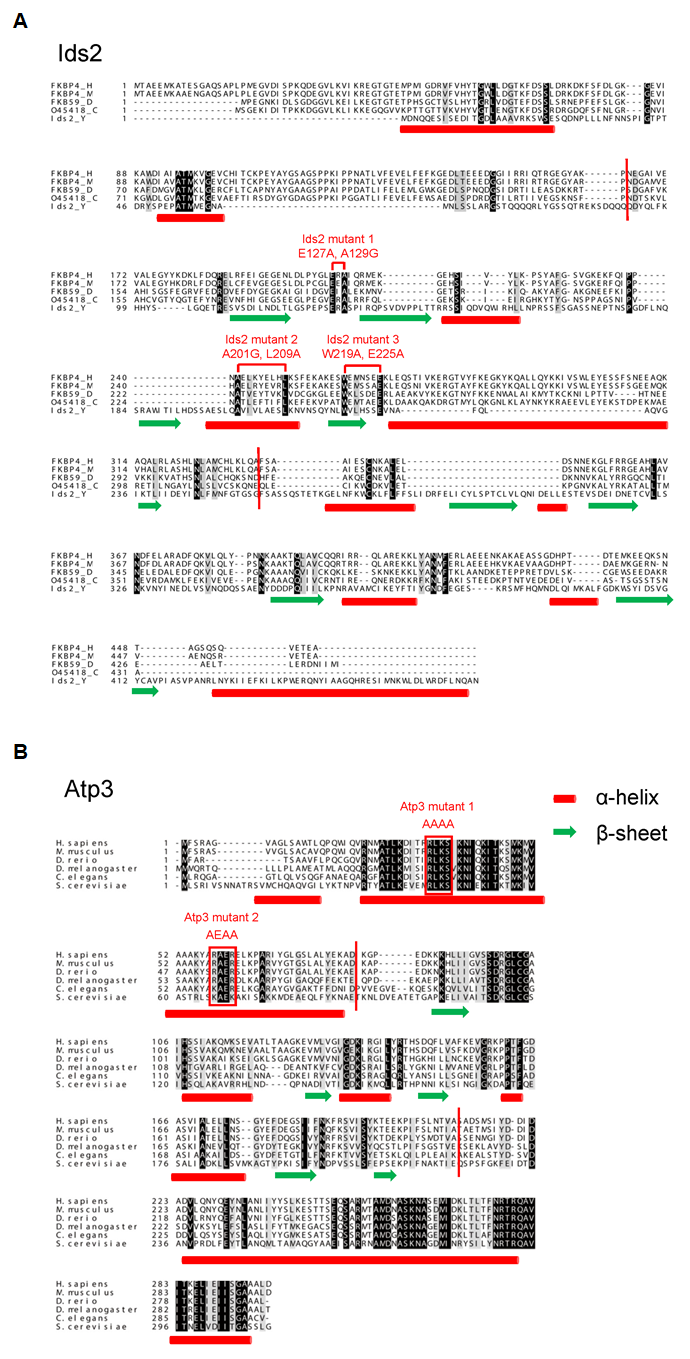
**

**Figure S5. Sequence alignments and secondary structure analyses of the Ids2’s and Atp3’s homologs.**

Sequence alignments of Ids2 **(A)** and Atp3 **(B)** with their predicted homologs were performed by the Clustal Omega program (https://www.ebi.ac.uk/Tools/msa/clustalo/). Conserved residues were coloured in black and practically conserved residues were coloured in grey. Secondary structures were predicted by the CFSSP program (http://www.biogem.org/tool/chou-fasman/). The α-helix and β-sheet were marked by red lines and green arrows below the amino acid sequences, respectively.


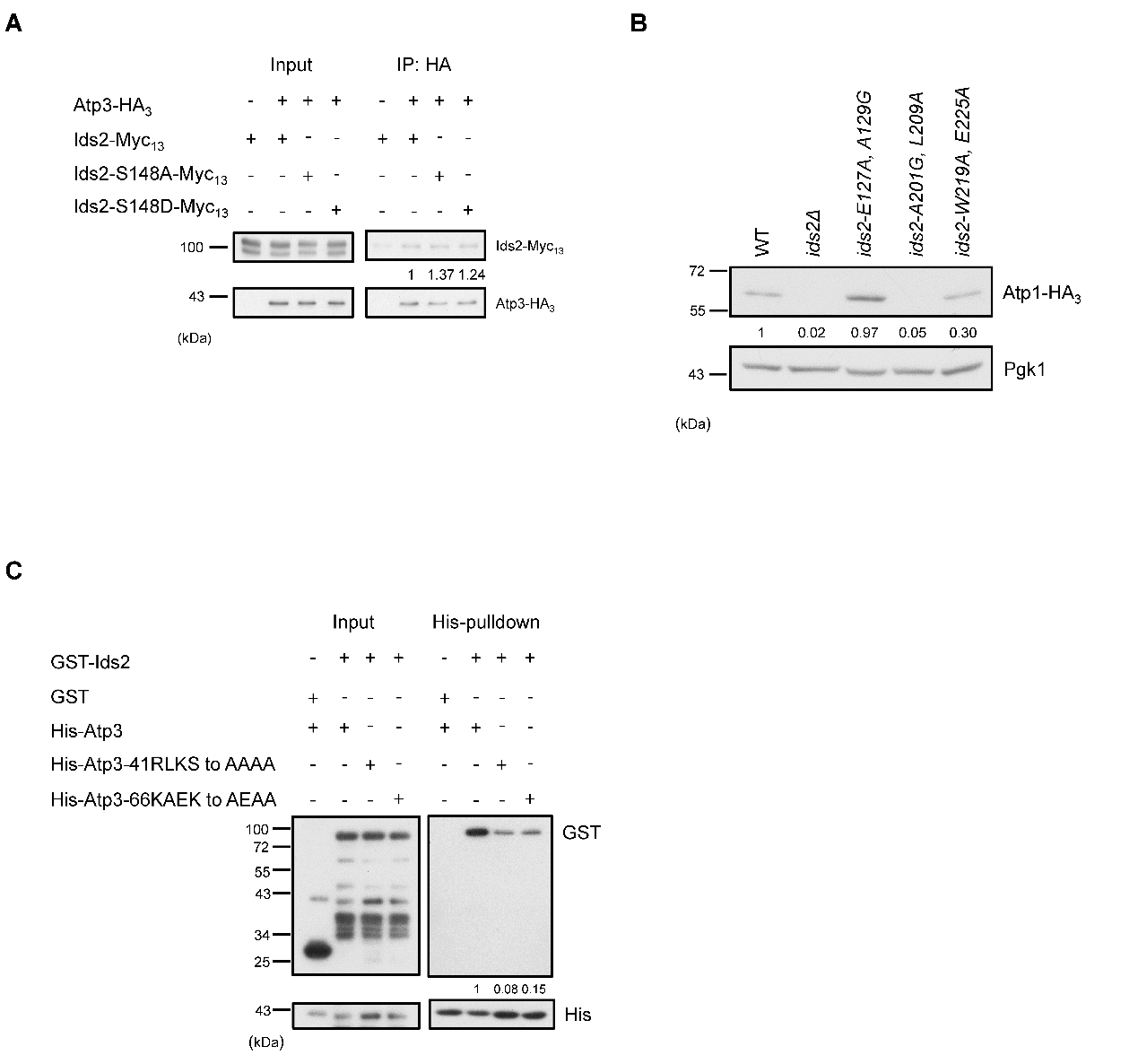


**Figure S6. The Ids2-Atp3 interaction and the Atp3 stability under various *ids2* mutant backgrounds.**

**(A)** Co-immunoprecipitation assays were conducted using cells transformed with the pRS423-Atp3-HA_3_ along with the pRS426-Ids2-Myc_13_, pRS426-S148A-Ids2-Myc_13_, or pRS426-S148D-Ids2-Myc_13_ plasmid. Lysates were precipitated by an HA antibody and co-precipitated proteins were detected by a Myc antibody. The numbers below are the intensity ratios of Myc/HA compared with that of the wild-type strain. **(B)** Indicated strains containing the pRS313-Atp1-HA_3_ plasmid were cultured overnight and then refreshed to OD = 0.5. Total protein was extracted, and the western blot was hybridized with an HA antibody. Pgk1 was served as a loading control. The numbers below are the intensity ratios of protein level/Pgk1 compared with that of the wild-type strains. **(C)** Talon-beads bound His_6_-Atp3 or His_6_-Atp3 mutants were incubated with full-length Ids2 recombinant proteins. Pulldown assay was conducted as described above. The numbers below are the intensity ratios of His-pulldown GST/His compared with that of the wild-type strain.


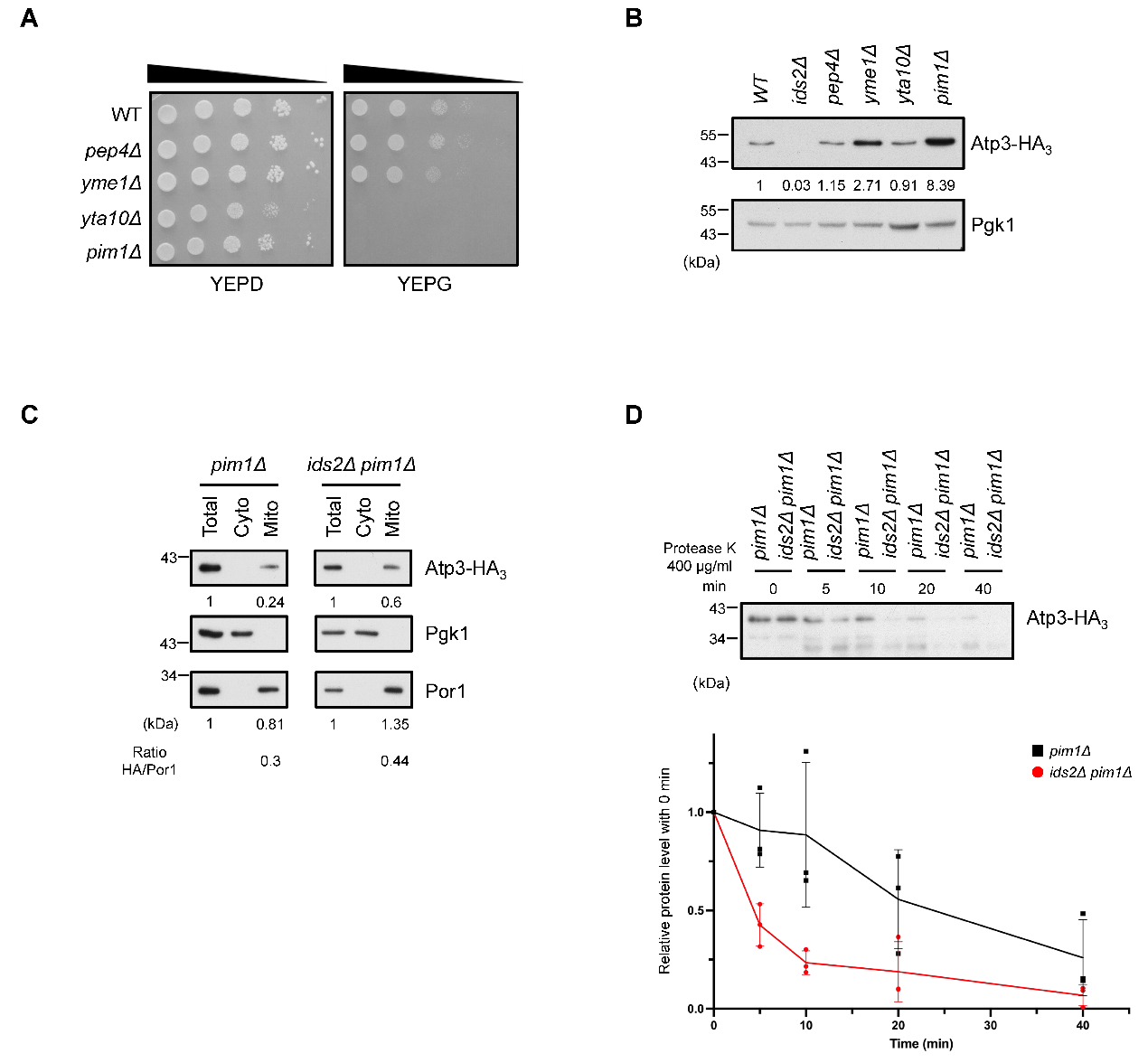


**Figure S7. Respiratory growth, Atp3 levels, Atp3 import, and Atp3 folding in various protease or *IDS2* deletional strains.**

**(A)** Ten-fold serially diluted cells were grown on SC plates supplemented with 2 % glucose or 3 % glycerol. **(B)** The protein level of Atp3-HA_3_ in the indicated strains was detected by western blotting. The numbers below are the intensity ratios of HA/Pgk1 compared with that of the wild-type strain. **(C)** Analysis of mitochondrial and cytosolic fractions. Yeast cells were treated with zymolyase and lysed with a Dounce homogenizer. Cellular compartments were fractionated to crude mitochondrial (Mito) and cytosolic fractions (Cyto) by differential centrifugation. Subcellular fractions were analyzed by Western blotting. Antibodies recognizing Atp3 (HA), cytosol (Pgk1), and mitochondria (Porin) were used. The numbers below are the intensity ratios of HA or Por1 compared with that of total protein extraction (Total). **(D)** W303 *pim1Δ* and *ids2Δ pim1Δ* strains were transformed with the pRS414-Atp3-HA_3_ plasmid. After a pull-down assay using anti-HA beads, Atp3-HA_3_ was subjected to limited proteolysis with 400 μg/ml Proteinase K for the indicated periods. The upper panel is the representative western blots. The lower panel is the line chart of full-length Atp3-HA_3_ (compared with the amount at time 0, mean ± SD, n = 3). Points on the line chart indicate individual data values from each biological replicate.


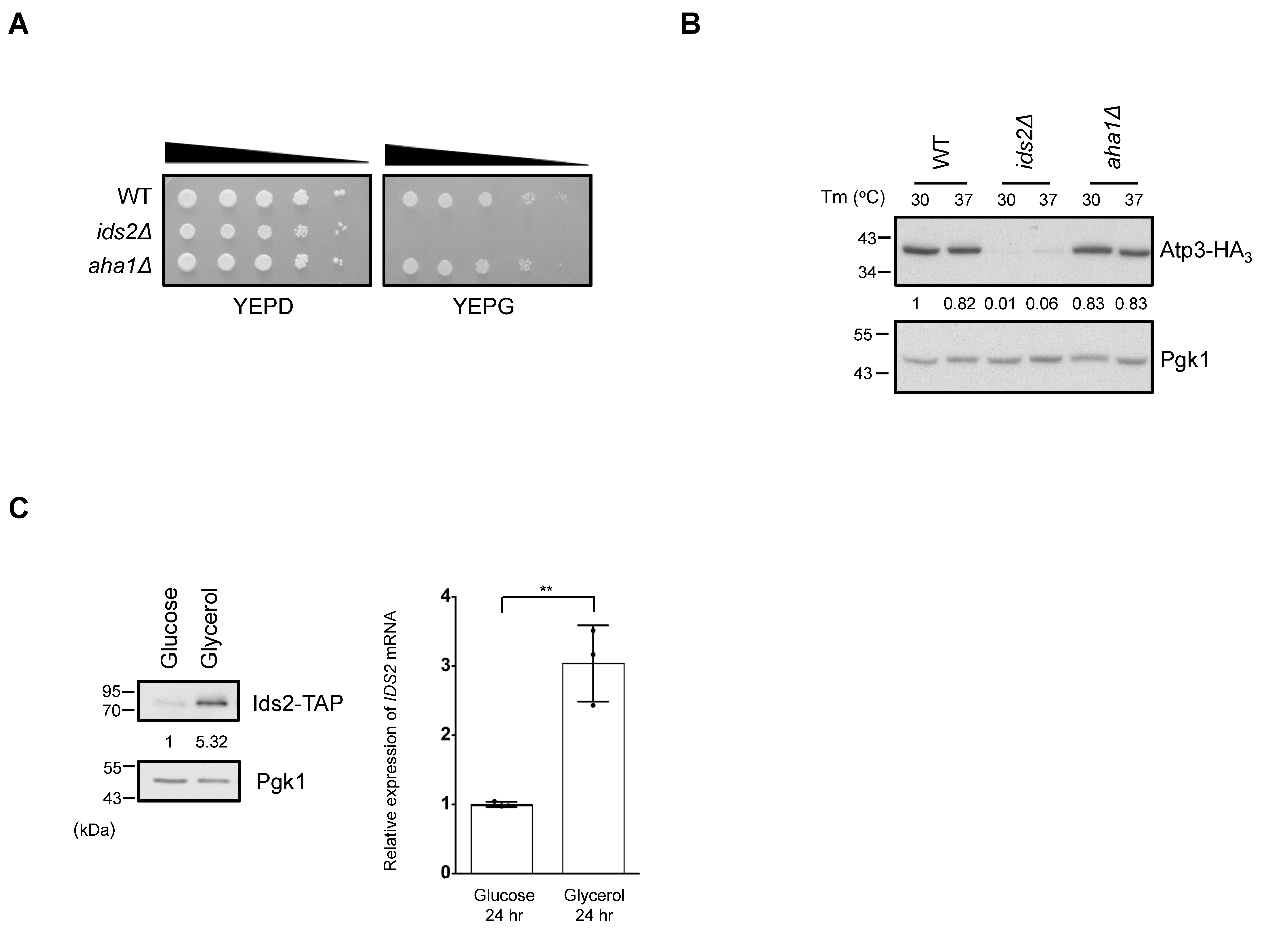


**Figure S8. Ids2 plays a dominant role for mitochondria under respiratory growth.**

**(A)** Ten-fold serially diluted cells were grown on SC plates supplemented with 2 % glucose or 3 % glycerol. **(B)** The protein level of Atp3-HA_3_ in the indicated strains was detected by western blotting. The numbers below are the intensity ratios of HA/Pgk1 compared with that of the wild-type strain. **(C)** Protein and mRNA levels of Ids2 under respiratory growth. Refreshed cells (OD = 0.5) were transferred to YEPD of YEPG for 24 hr. Protein levels detected by western blotting are shown at the left panel, and mRNA levels identified by RT-PCR are shown at the right. The numbers below are the intensity ratios of TAP/Pgk1 compared with that of cells grown in glucose. The values RT-PCR are mean ± SD (n = 3) and compared by using the Student’s t-test. **, *p-*value < 0.01. Points on bar graphs indicate individual data values from each biological replicate.


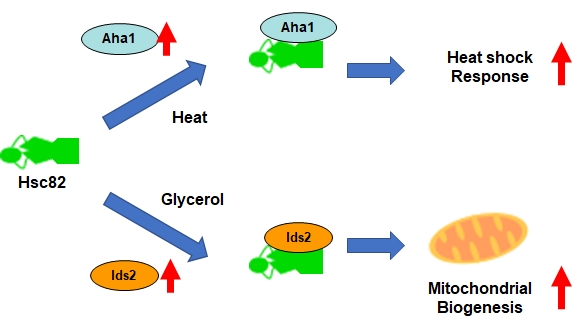


**Figure S9. A proposed model describes that two Hsc82 cochaperones, Ids2 and Aha1, may split their works under different environmental conditions.**
