# Supplementary material for: An HSP90 cochaperone Ids2 maintains the stability of mitochondrial DNA and ATP synthase
Source: BMC Biol. 2021 Nov 11;19:242. doi: 10.1186/s12915-021-01179-x (PMC8582188; doi:10.1186/s12915-021-01179-x)
Supplement: Supplementary file 5 — Additional file 5. Images of the full immunoblots. [file 12915_2021_1179_MOESM5_ESM.pdf]

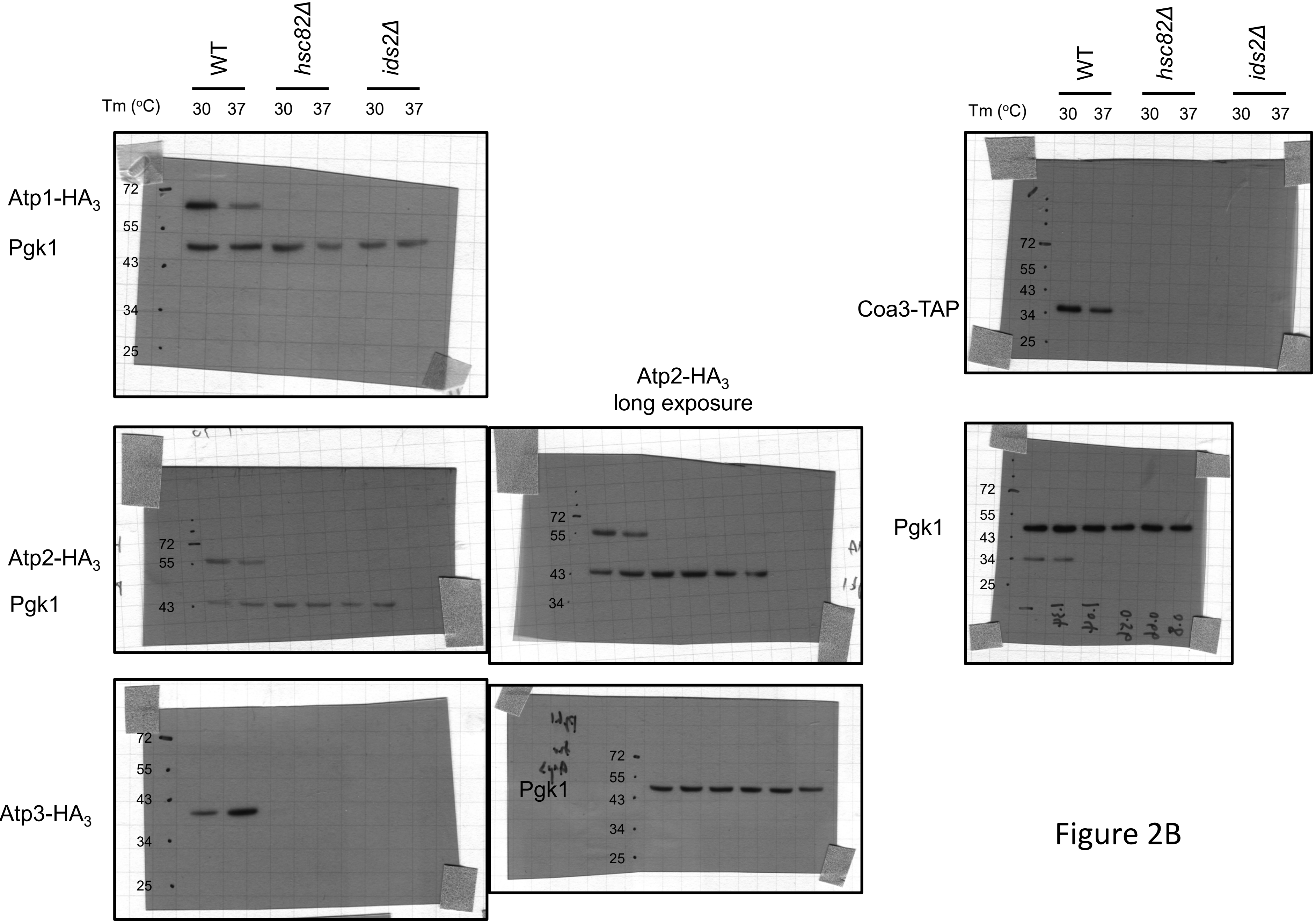

WT
hsc82Δ
ids2Δ
Tm (°C)
30 37 30 37 30 37
Atp1-HA3
Pgk1
72
55
43
34
25
Atp2-HA3
Pgk1
Atp2-HA3
long exposure
Atp3-HA3
Coa3-TAP

Figure 2B

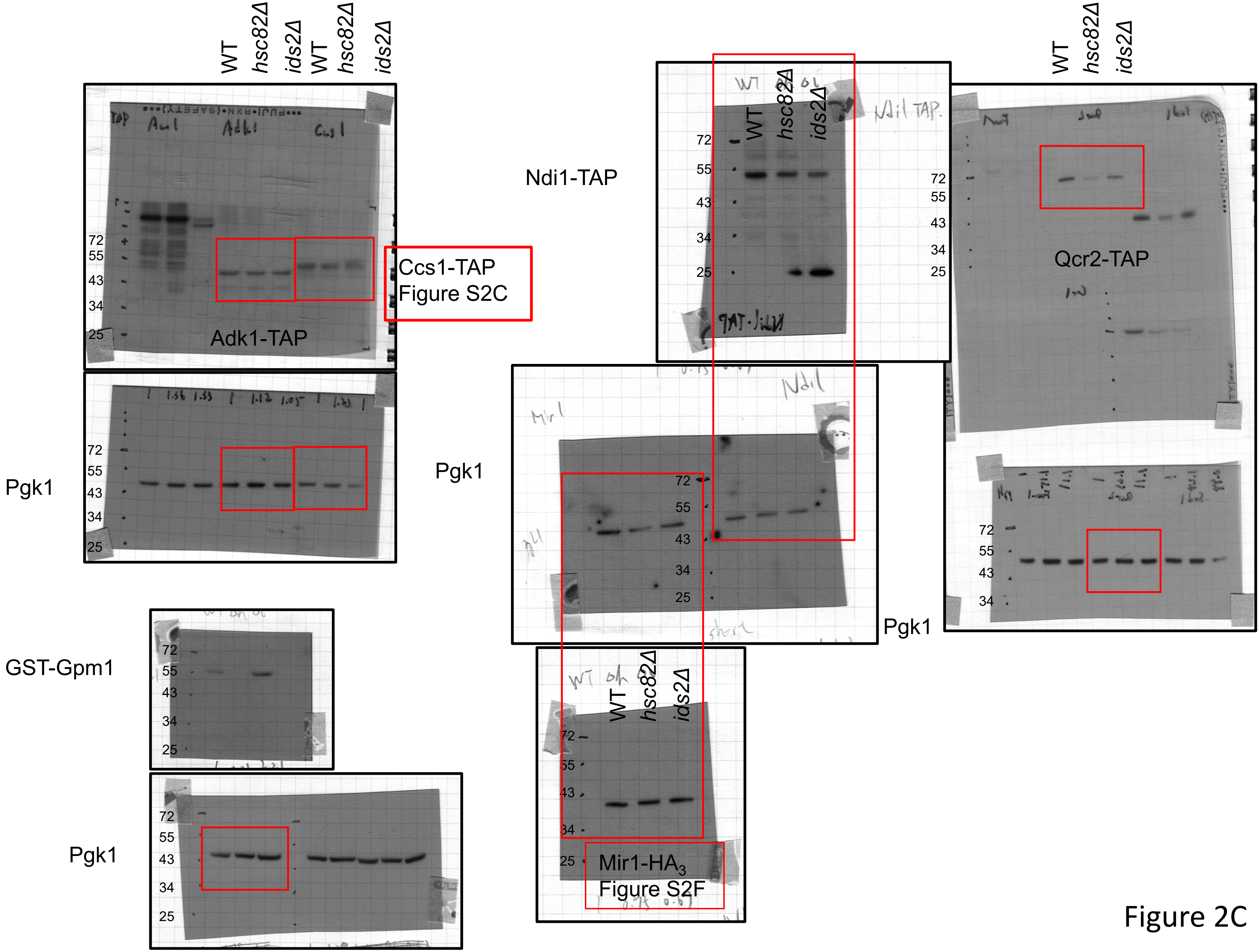
WT hsc82Δ ids2Δ WT hsc82Δ ids2Δ
Ccs1-TAP
Figure S2C
Adk1-TAP
72
55
43
34
25
Pgk1
GST-Gpm1
Pgk1
Ndi1-TAP
WT hsc82Δ ids2Δ
Pgk1
Pgk1
WT hsc82Δ ids2Δ
Mir1-HA3
Figure S2F
WT hsc82Δ ids2Δ
Qcr2-TAP
Pgk1

Figure 2C

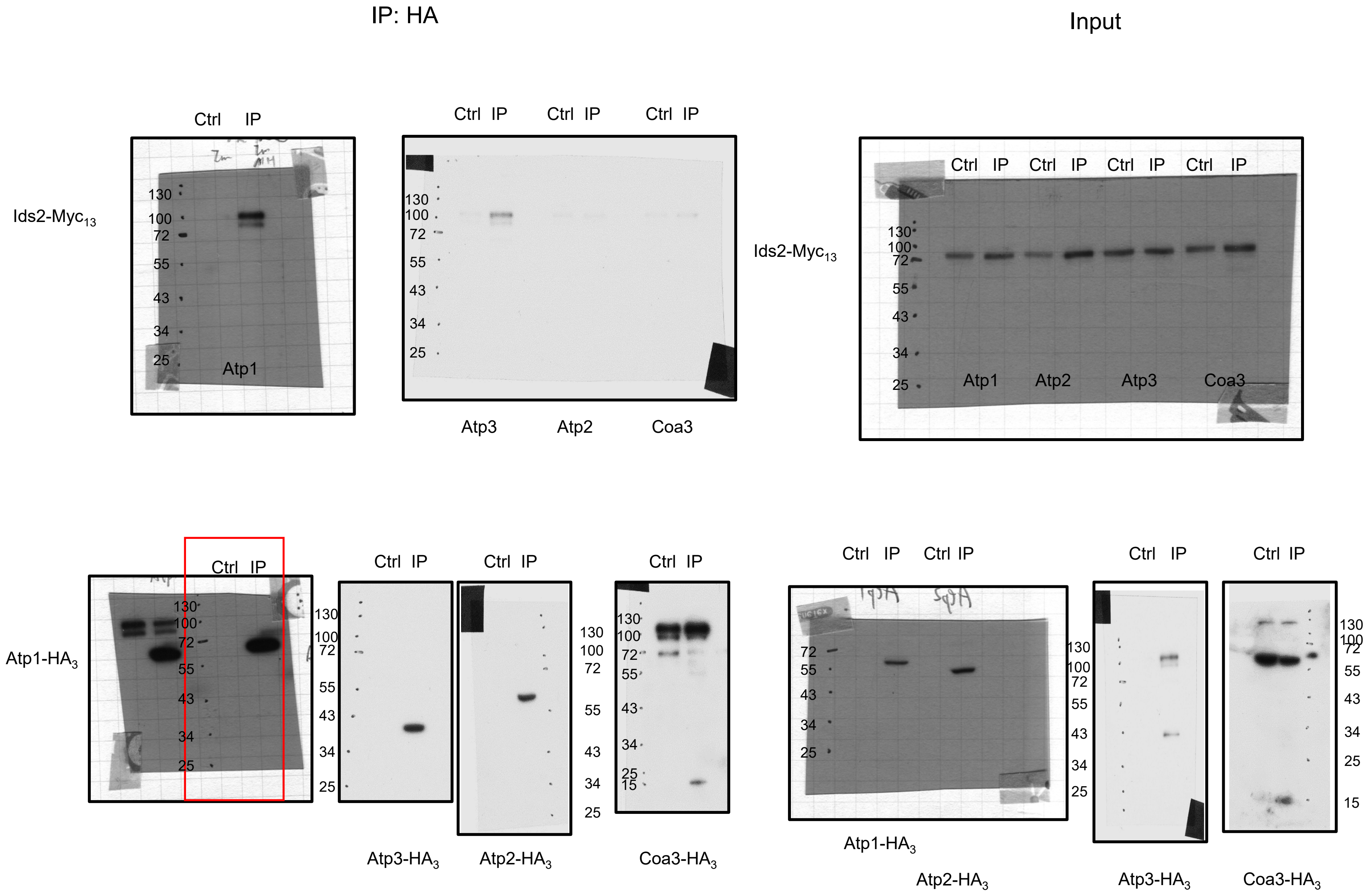
IP: HA
Input
Ctrl IP
Ctrl IP Ctrl IP Ctrl IP
Ctrl IP Ctrl IP Ctrl IP Ctrl IP
Ids2-Myc13
Atp1
Atp3
Atp2
Coa3
Atp1 Atp2 Atp3 Coa3
130
100
72
55
43
34
25
15
Atp1-HA3
Atp3-HA3
Atp2-HA3
Coa3-HA3

Figure 4A

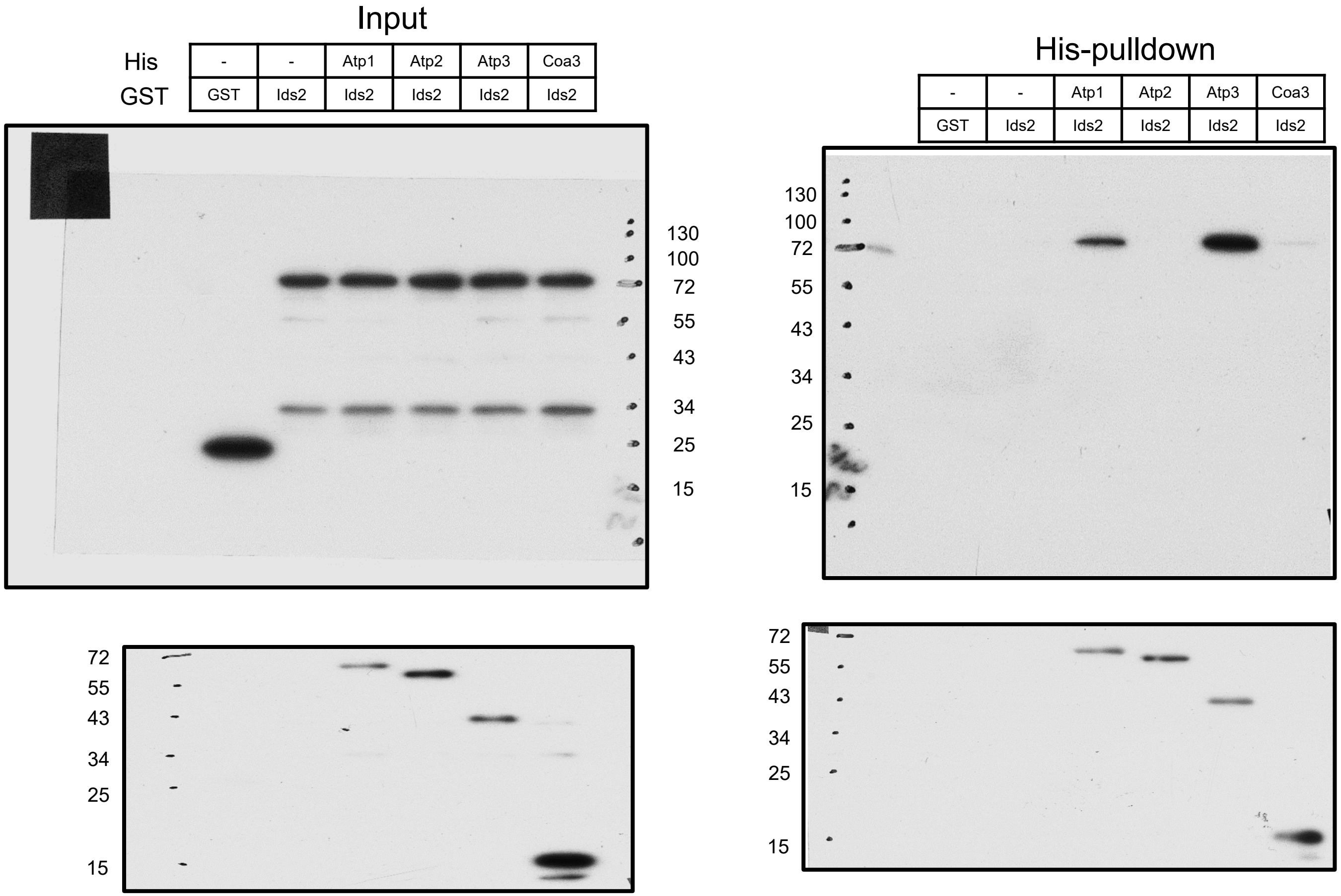
Input
His
GST
- - Atp1 Atp2 Atp3 Coa3
GST Ids2 Ids2 Ids2 Ids2 Ids2
130
100
72
55
43
34
25
15
His-pulldown
- - Atp1 Atp2 Atp3 Coa3
GST Ids2 Ids2 Ids2 Ids2 Ids2
130
100
72
55
43
34
25
15
72
55
43
34
25
15
72
55
43
34
25
15

Figure 4B

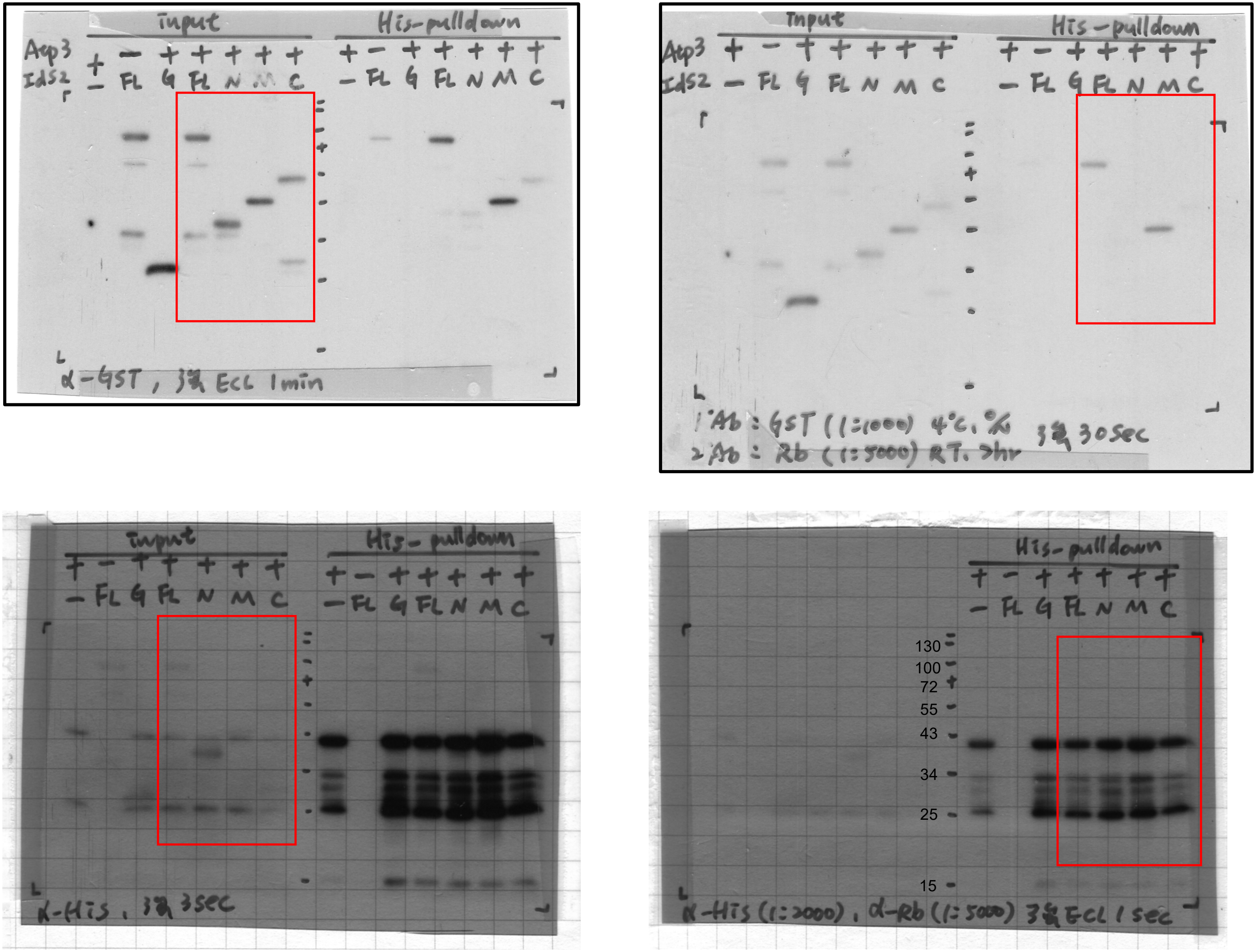

Input
His-pulldown
α-GST, 3% ECL 1min
α-His, 3% 3sec
130
100
72
55
43
34
25
15
α-His (1:2000), α-Rb (1:5000) 3% ECL 1sec

Figure 5A

GST

His

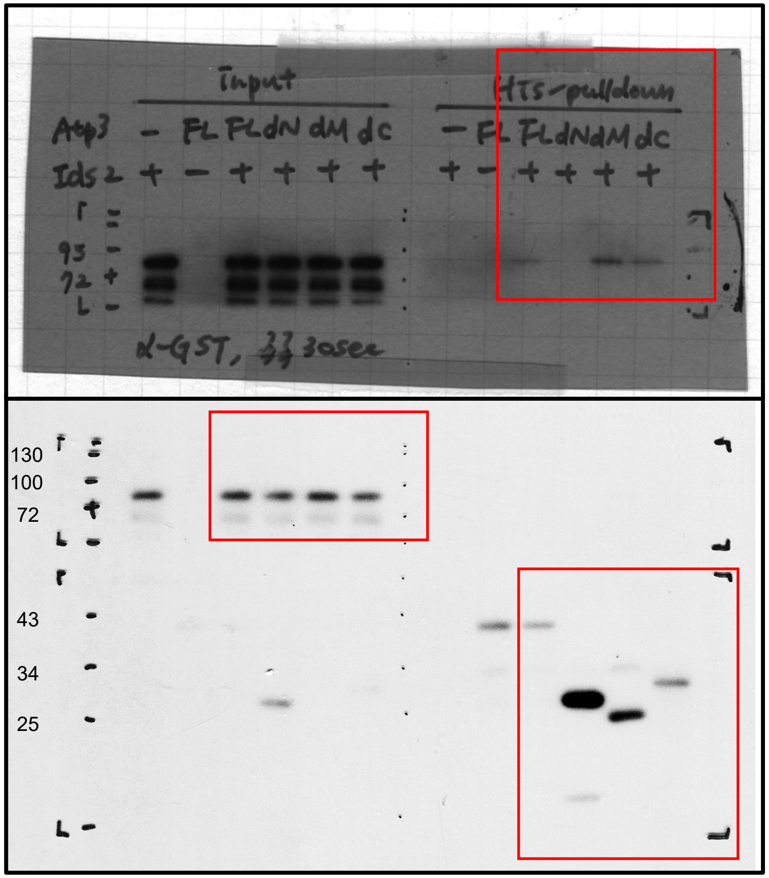

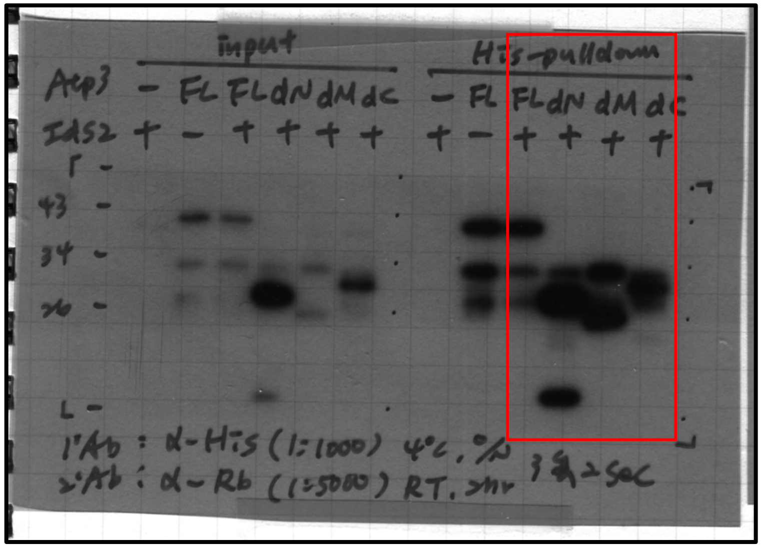

Figure 5C

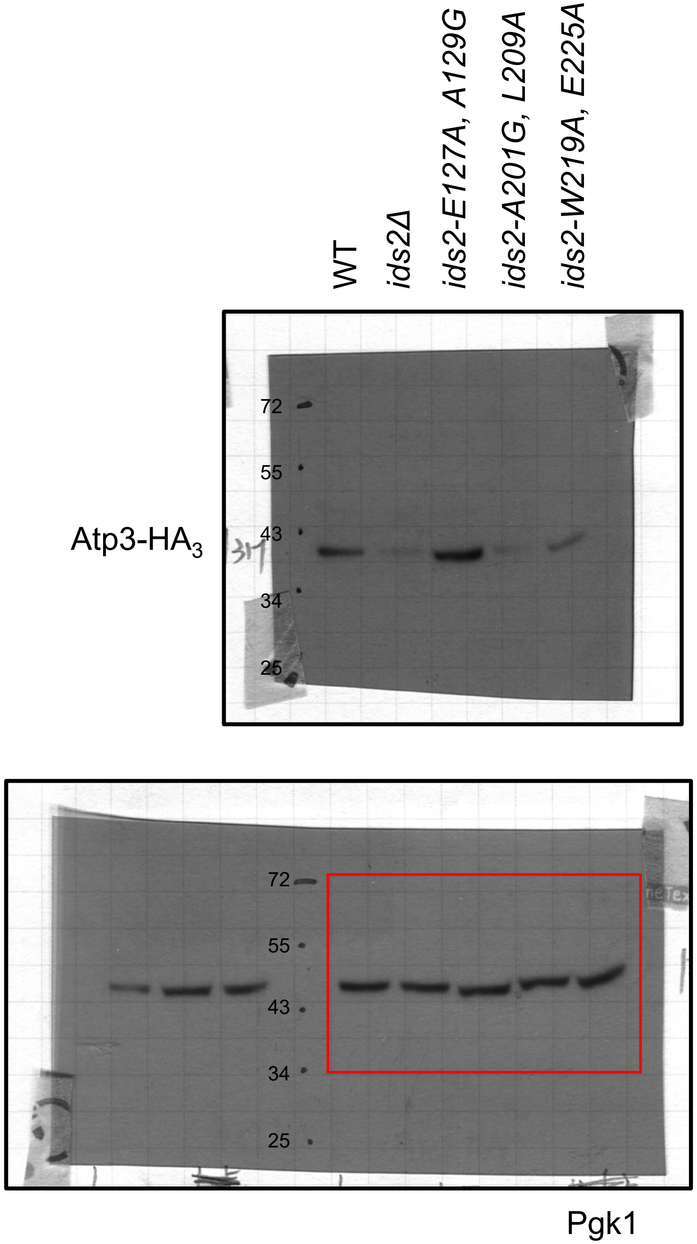

Figure 6B

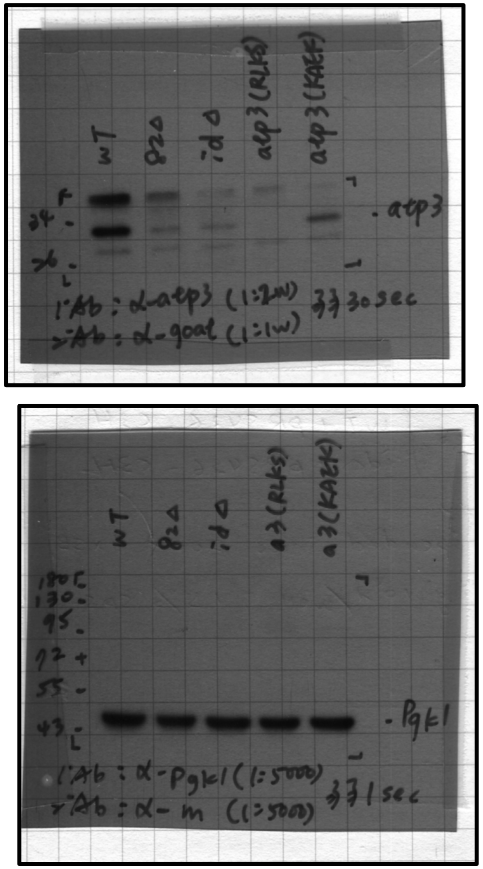

Figure 6D

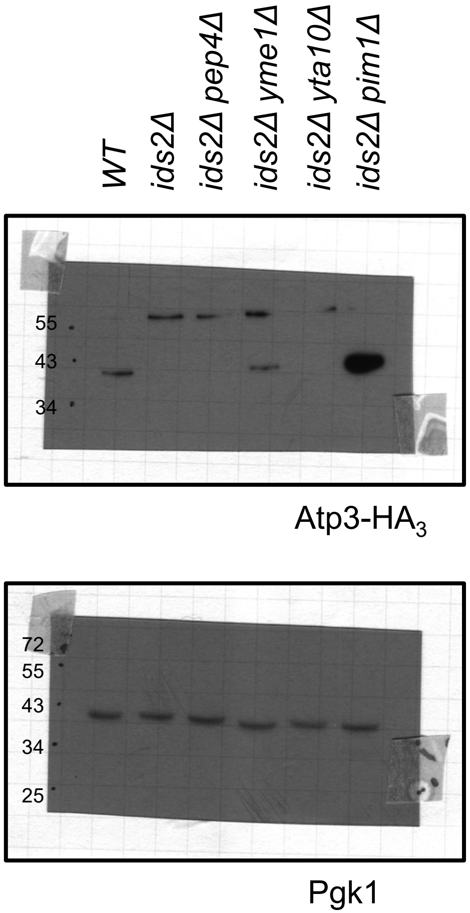

Figure 7B

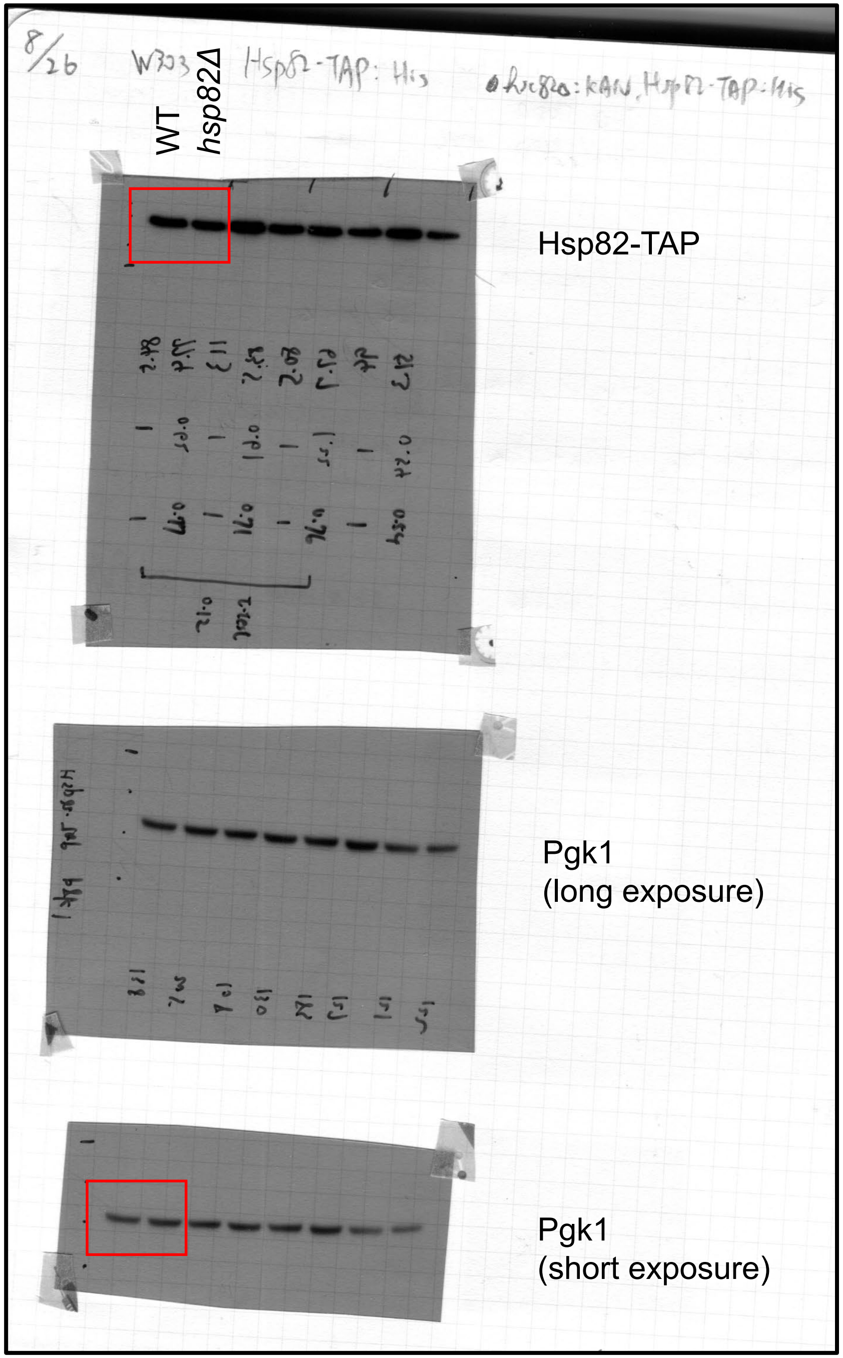

Figure S1B

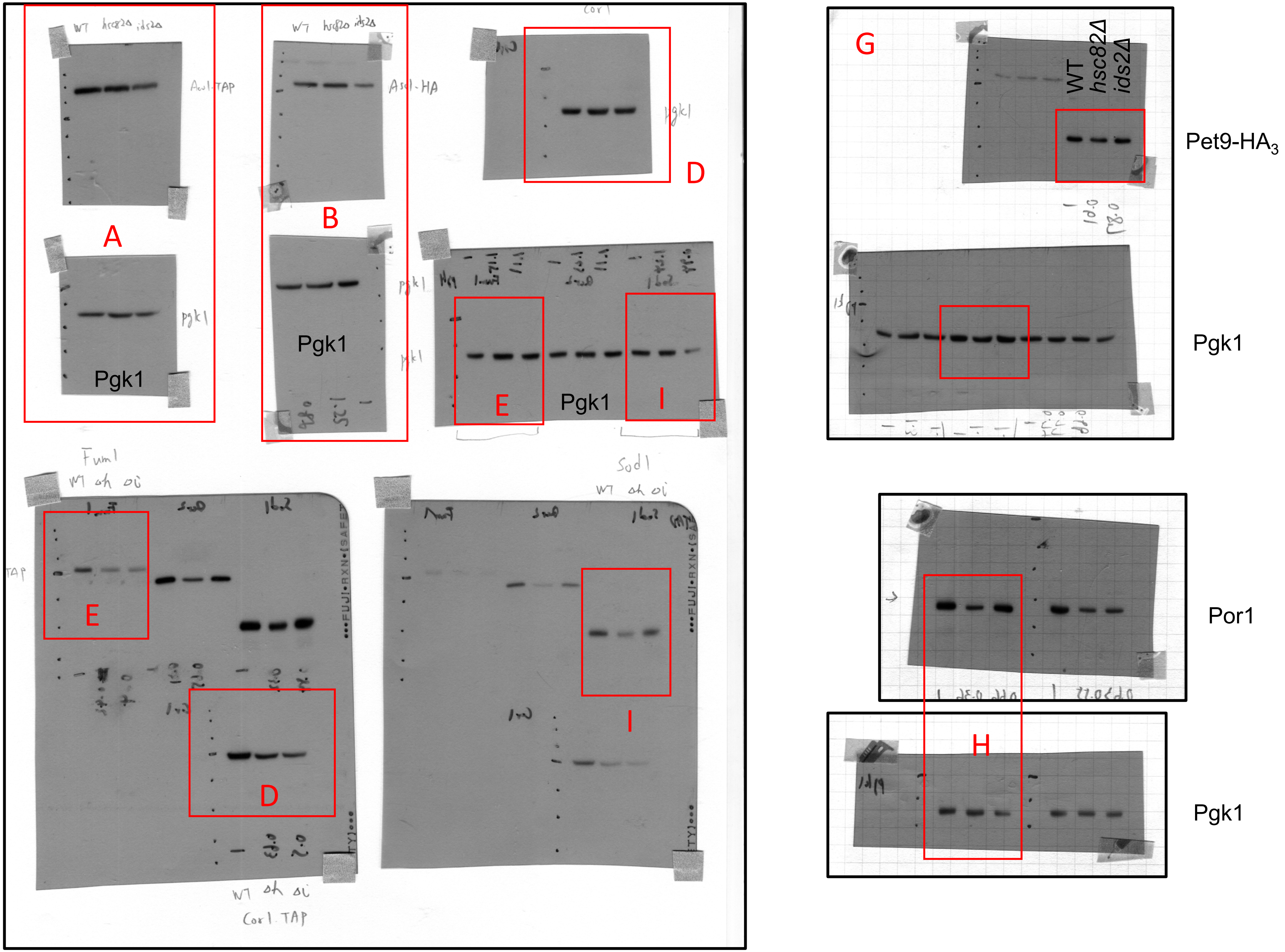
A
Pgk1
B
Pgk1
D
E
Pgk1
I
E
D
I
G
WT
hsc82Δ
ids2Δ
Pet9-HA3
Pgk1
Por1
H
Pgk1

Figure S2

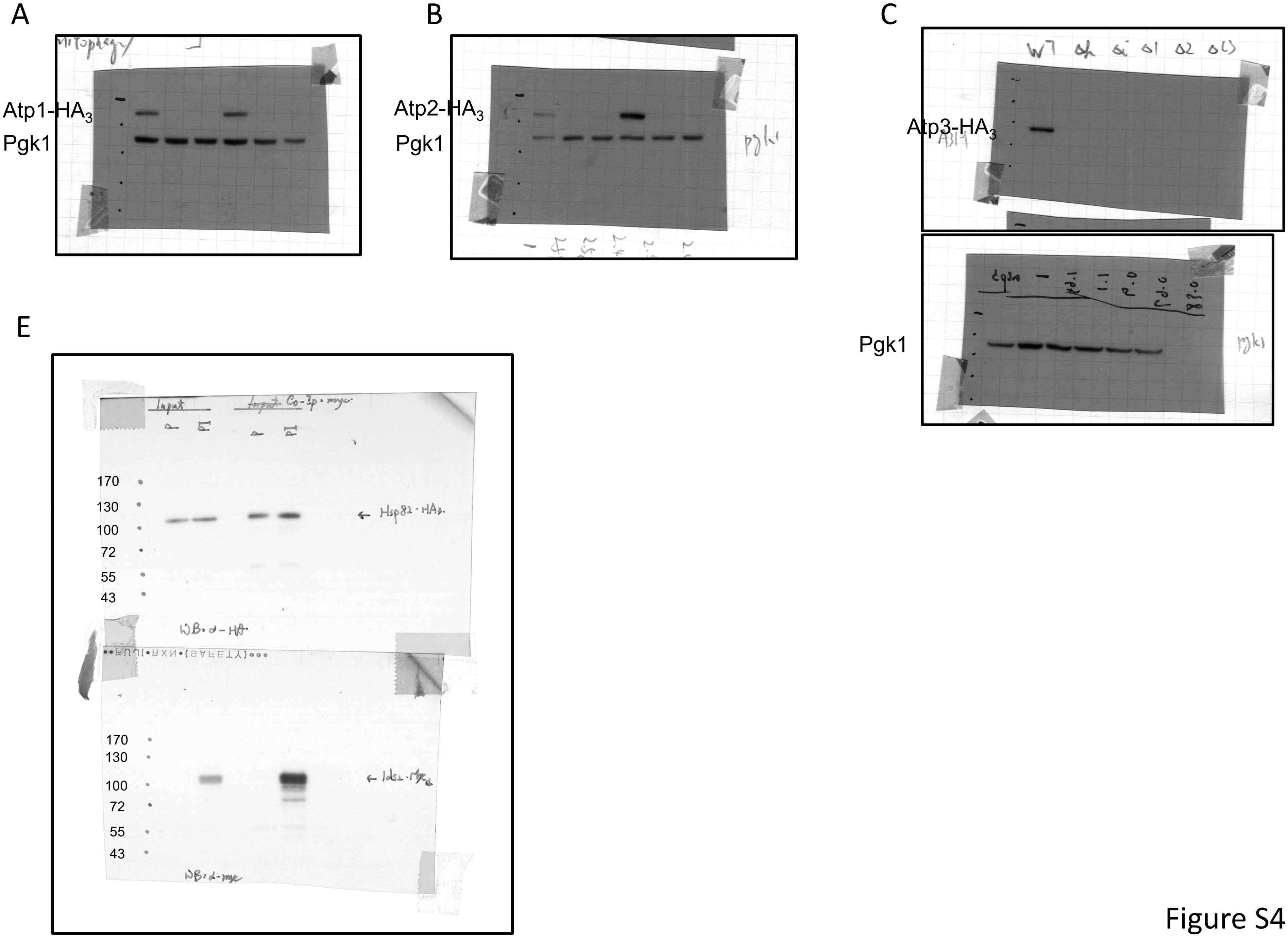
A
Atp1-HA3
Pgk1
B
Atp2-HA3
Pgk1
C
Atp3-HA3
Pgk1
E
170
130
100
72
55
43
Hsp82-HA3
WB: α-HA
170
130
100
72
55
43
WB: α-myc

Figure S4

D

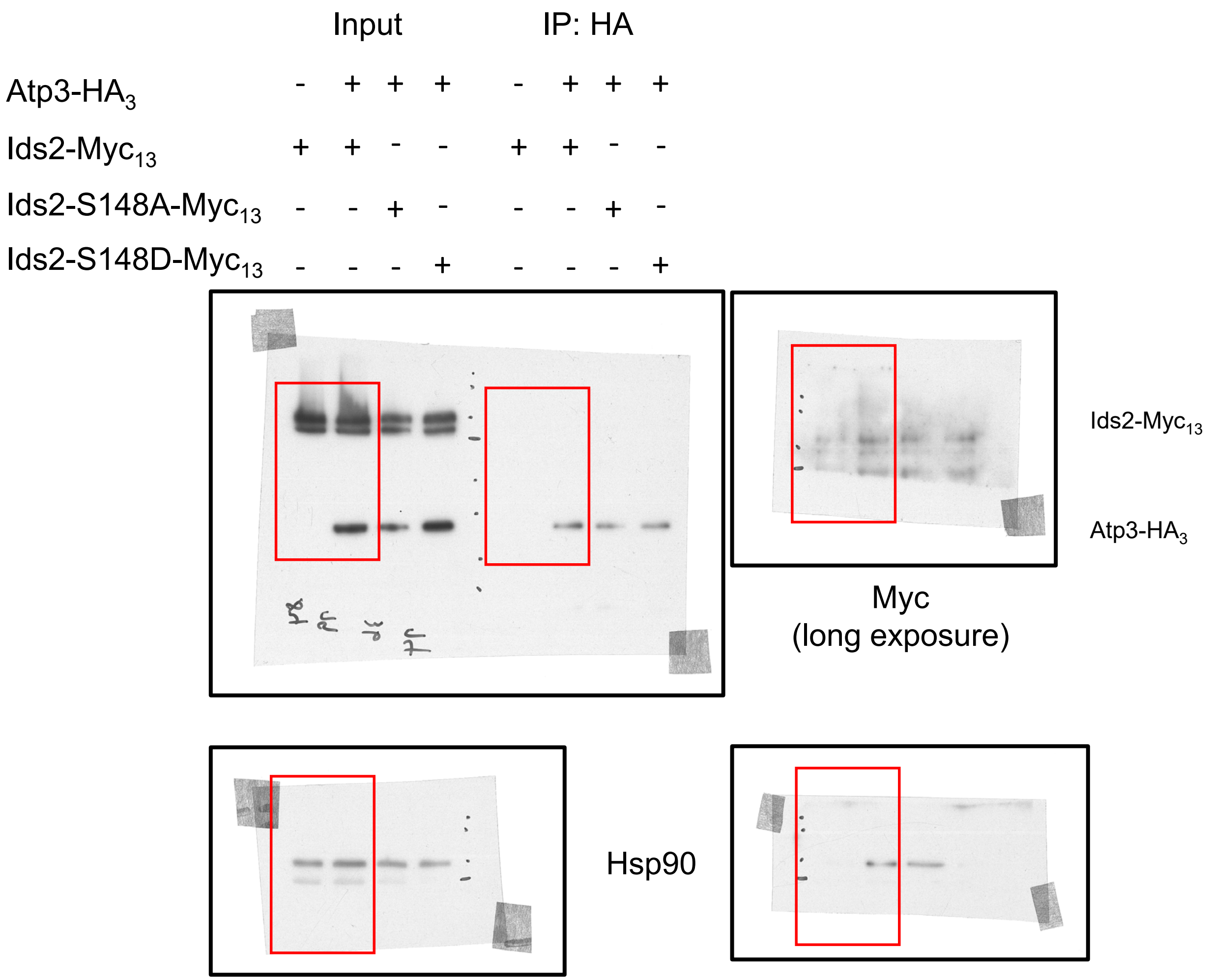
Input
IP: HA
Atp3-HA3 - + + + - + + +
Ids2-Myc13 + + - - + + - -
Ids2-S148A-Myc13 - - + - - - + -
Ids2-S148D-Myc13 - - - + - - - +
Ids2-Myc13
Atp3-HA3
Myc
(long exposure)
Hsp90

Figure S4

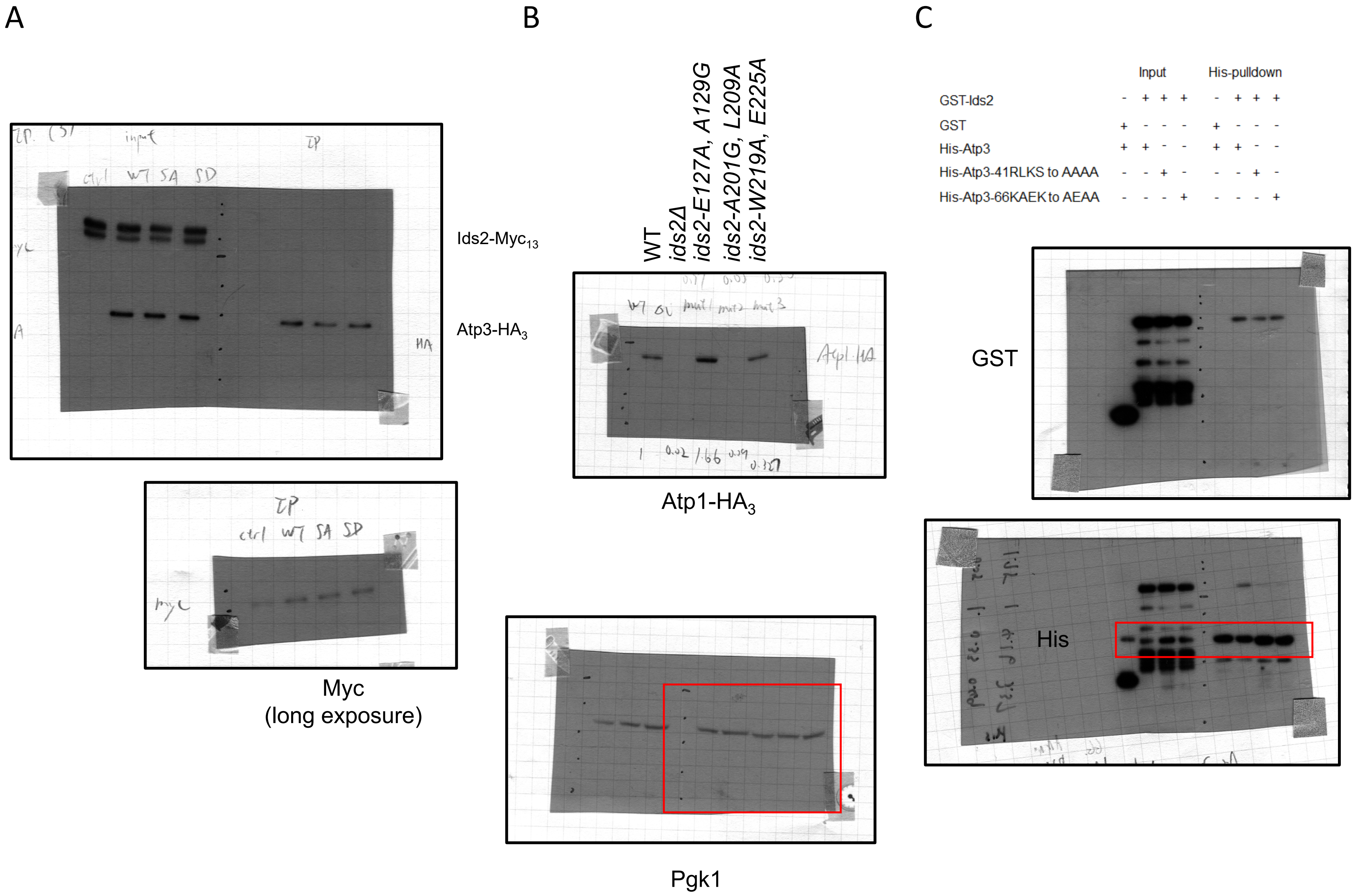
A
B
C
Ids2-Myc13
Atp3-HA3
Myc
(long exposure)
WT
ids2Δ
ids2-E127A, A129G
ids2-A201G, L209A
ids2-W219A, E225A
Atp1-HA3
Pgk1
Input
His-pulldown
GST-Ids2
GST
His-Atp3
His-Atp3-41RLKS to AAAA
His-Atp3-66KAEK to AEAA
GST
His

Figure S6

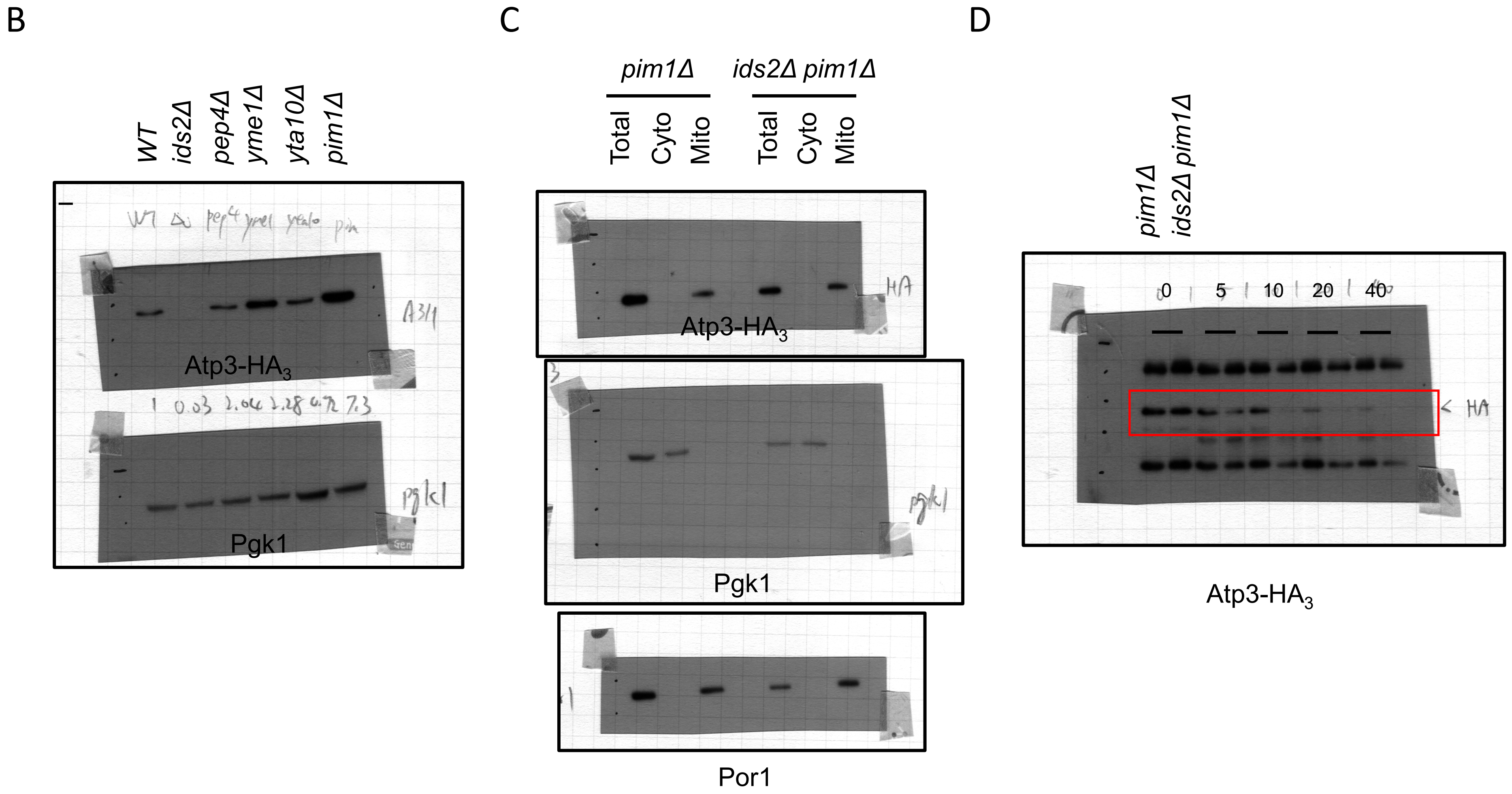
B
WT
ids2Δ
pep4Δ
yme1Δ
yta10Δ
pim1Δ
Atp3-HA3
Pgk1
C
pim1Δ
ids2Δ pim1Δ
Total
Cyto
Mito
Total
Cyto
Mito
Atp3-HA3
Pgk1
Por1
D
pim1Δ
ids2Δ pim1Δ
0
5
10
20
40
Atp3-HA3

Figure S7

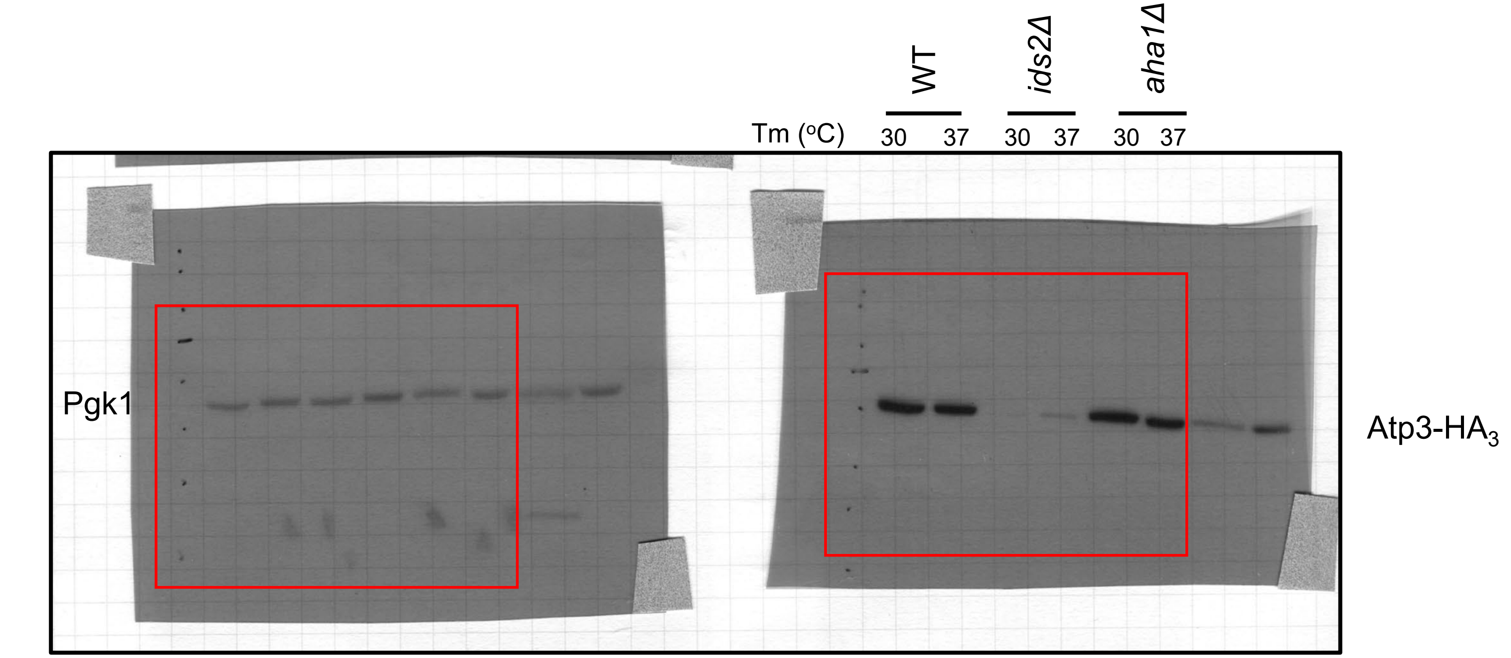

Figure S8B

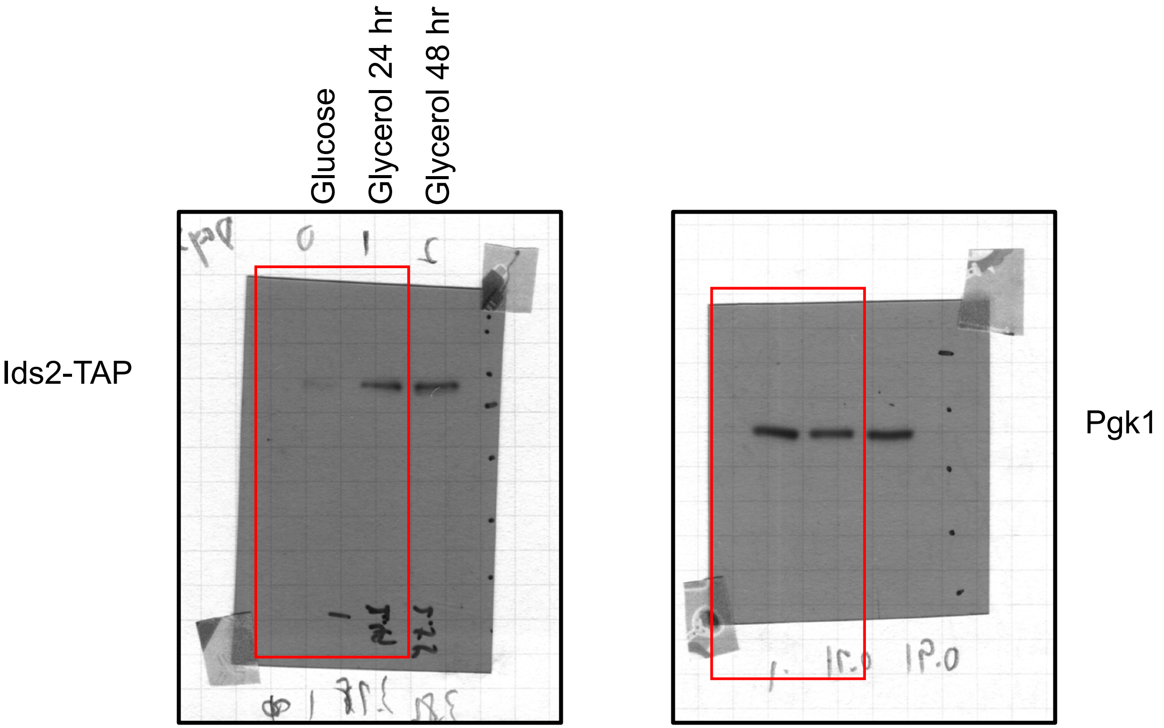

Figure S8C
